# *In silico* approaches for designing highly effective cell penetrating peptides

**DOI:** 10.1186/1479-5876-11-74

**Published:** 2013-03-22

**Authors:** Ankur Gautam, Kumardeep Chaudhary, Rahul Kumar, Arun Sharma, Pallavi Kapoor, Atul Tyagi, Open source drug discovery consortium, Gajendra P S Raghava

**Affiliations:** 1 Bioinformatics Centre, CSIR-Institute of Microbial Technology, Chandigarh 160036, India; 2 Open Source Drug Discovery Consortium, Council of Scientific and Industrial Research (CSIR), Anusandhan Bhawan, 2 Rafi Marg, New Delhi 110001, India

**Keywords:** Cell penetrating peptides, Drug delivery, Amino acid composition, Support vector machine

## Abstract

**Background:**

Cell penetrating peptides have gained much recognition as a versatile transport vehicle for the intracellular delivery of wide range of cargoes (*i.e.* oligonucelotides, small molecules, proteins, *etc.*), that otherwise lack bioavailability, thus offering great potential as future therapeutics. Keeping in mind the therapeutic importance of these peptides, we have developed *in silico* methods for the prediction of cell penetrating peptides, which can be used for rapid screening of such peptides prior to their synthesis.

**Methods:**

In the present study, support vector machine (SVM)-based models have been developed for predicting and designing highly effective cell penetrating peptides. Various features like amino acid composition, dipeptide composition, binary profile of patterns, and physicochemical properties have been used as input features. The main dataset used in this study consists of 708 peptides. In addition, we have identified various motifs in cell penetrating peptides, and used these motifs for developing a hybrid prediction model. Performance of our method was evaluated on an independent dataset and also compared with that of the existing methods.

**Results:**

In cell penetrating peptides, certain residues (*e.g.* Arg, Lys, Pro, Trp, Leu, and Ala) are preferred at specific locations. Thus, it was possible to discriminate cell-penetrating peptides from non-cell penetrating peptides based on amino acid composition. All models were evaluated using five-fold cross-validation technique. We have achieved a maximum accuracy of 97.40% using the hybrid model that combines motif information and binary profile of the peptides. On independent dataset, we achieved maximum accuracy of 81.31% with MCC of 0.63.

**Conclusion:**

The present study demonstrates that features like amino acid composition, binary profile of patterns and motifs, can be used to train an SVM classifier that can predict cell penetrating peptides with higher accuracy. The hybrid model described in this study achieved more accuracy than the previous methods and thus may complement the existing methods. Based on the above study, a user- friendly web server CellPPD has been developed to help the biologists, where a user can predict and design CPPs with much ease. CellPPD web server is freely accessible at http://crdd.osdd.net/raghava/cellppd/.

## Background

Poor delivery and low bioavailability of therapeutic molecules are the two main obstacles in the drug development process. The plasma membrane is selectively permeable and remains a major barrier for most of the therapeutic molecules. In order to overcome this barrier, a number of delivery systems have been developed over the years [1,2]. Despite the tremendous progress, the existing delivery methods can result in high toxicity, immunogenicity and low delivery yield. In the last decade, short peptides known as cell penetrating peptides (CPPs) or protein transduction domains (PTDs) have gained much recognition as an efficient delivery vehicle [3]. CPPs have remarkable ability to transverse eukaryotic membranes without significant membrane damage. In addition, CPPs can carry a variety of cargoes like peptides [4,5], proteins [6], drugs [7,8], nucleic acids [9,10], siRNAs [11,12], nanoparticles [13,14], *etc.* across the cell membrane. Almost everything can be transported into the cell, once conjugated to CPP [15]. Thus, CPPs have a great therapeutic potential, especially in drug delivery. Although first CPP has been discovered 25 years ago, their mechanism of uptake is still not very clear. However, two routes of internalization have been proposed that include direct penetration and endocytic pathway [16].

Since the discovery of first CPP, *i.e.* Tat (transcription activator of the human immunodeficiency virus type 1) peptide, hundreds of CPPs have been discovered so far with varied length and physicochemical properties [17]. Most of these peptides are short (up to 35 amino acids), water soluble, partly hydrophobic, and/or polybasic in nature with a net positive charge at physiological pH [18]. In the past, few attempts have been made to develop computational methods for CPP prediction [19-22]. In 2008, Hansen *et al.* developed a method, which involves a set of z-scales of 87 coded and non-coded amino acids published by Sandberg and his group [23]. z-scales require a lot of variables like molecular weight, molecular orbital calculations, proton NMR shift, *etc.* Finally, z-scores obtained are used to predict the CPPs. This method gave 68% prediction efficiency, which is very poor to distinguish CPPs from the non-CPPs. In 2010, Dobchev *et al.* used quantitative structure-activity relationship (QSAR) and artificial neural network (ANN) models to predict CPPs. They achieved maximum accuracy of 83%. In this method, sequences that are difficult to predict were excluded. In a recent study, Sanders *et al.* (2011) have used support vector machine (SVM) models to predict CPPs on five different datasets. They used various biochemical properties to develop SVM models. One of the major limitations with the previous methods is that datasets used for training were very small (< 111) and none of the methods is available in the form of web service for public use. In addition, most of the previous methods have used unbalanced datasets, which presents many problems for machine learning classifiers. This point has also been highlighted earlier by Sanders *et al.* in their study, where they have used both balanced and unbalanced datasets for machine learning. In balanced dataset, they achieved 95% accuracy and 75% accuracy was achieved in unbalanced dataset. This poor performance of SVM with unbalanced dataset is due to the inherent learning biases of unbalanced dataset, demonstrating the need for balanced datasets for avoiding biases in machine learning.

In the present study, we have made a systematic attempt to complement existing methods for predicting CPPs with high accuracy. We have used large dataset (708 CPPs) for training, testing and evaluating our models. The dataset is derived from the CPPsite, which is the first database of experimentally validated CPPs [24]. We have used various features like amino acid composition, dipeptide composition, binary profiles of pattern, and physicochemical properties as input for developing SVM models. In addition, we have also identified various CPP specific motifs, which have been used to develop a hybrid model. For the first time, a prediction web tool has been developed to assist the scientific community working in the area of CPPs.

## Methods

### Main datasets

We have extracted 843 experimentally validated CPPs from the CPPsite database, which has been developed by our group [24]. The peptides containing non-natural amino acids (*e.g.* selenocysteine) or having D-amino acids (D-conformation) were removed. Finally, we have got 708 unique CPPs having natural amino acids. Three different datasets (CPPsite-1, CPPsite-2 and CPPsite-3) have been created from these peptides. Since very few peptides have been experimentally validated as non-CPPs (negative examples), equal number of peptides (15–30 amino acids) were generated randomly from SwissProt proteins, and considered them as non-CPPs. This strategy for creating negative dataset has already been used in previous studies [22,25].

First dataset (CPPsite-1) contains 708 CPPs (positive examples) and 708 non-CPPs (negative examples). In CPPsite-1, CPPs having wide range of uptake efficiency (low and high) have been included, thus we have derived another dataset CPPsite-2 from CPPsite-1. CPPsite-2 contains 187 CPPs having high uptake efficiency and equal number of non-CPPs. We have created third dataset (CPPsite-3), which contains 187 CPPs having high uptake efficacy as positive examples and equal number of CPPs with low uptake efficiency were taken as negative examples. The model based on CPPsite-3 dataset can discriminate between high and low efficient CPPs.

All datasets (CPPsite-1, CPPsite-2 and CPPsite-3) consist of several CPPs with all possible Ala-scan mutants, or different truncations. Ideally redundancy in the datasets should be removed because it affects the performance of prediction method. In past, our group has removed the redundancy in various prediction methods [25,26]. But in this study, we have not removed the redundancy in CPP datasets because a single residue can affect the uptake efficiency of CPPs, and this may also lead to the loss of information about CPPs. In order to check the performance of our model on redundant dataset, we have used some benchmark datasets, which are redundant.

### Benchmark datasets

In order to compare our method with existing methods, we have extracted datasets from literature that have been used in previous studies. Sanders *et al.* (2011) have developed a method for CPP prediction. In this study, they have used 111 experimentally validated CPPs and equal number of non-CPPs (generated randomly from the chicken proteome). We have named this dataset Sanders-2011a. Second dataset from Sanders *et al.* (2011) named Sanders-2011b, which contains 111 CPPs and 34 experimentally validated non-CPPs. We have also generated a third dataset Sanders-2011c consisting of 111 CPPs, and 111 non-CPPs randomly sampled from 34 known non-CPPs. Dobchev *et al.* (2010) have used 74 CPPs and 24 non-CPPs for developing method for CPP prediction. These peptides were collected from the literature. We have used this dataset in this study and named Dobchev-2010. Similarly, we have created datasets Hansen-2008 (containing 66 CPPs & 19 non-CPPs) [20] and Hallbrink-2005 (containing 53 CPPs & 16 non-CPPs) from previous studies [19].

### Independent dataset

In order to evaluate the performance of our method, we have created an independent dataset of 99 novel CPPs, which have not been included in the training, feature selection and parameter optimization of the model. These peptides have been collected manually from recent research papers and patents.

### Cross-validation technique

The validation of any prediction method is very essential part. In the present study, five-fold cross-validation technique was used to evaluate the performance of all the models. Here, sequences are randomly divided into five sets, of which four sets are used for training and the remaining fifth set for testing. The process is repeated five times in such a way that each set is used once for testing. Final performance is obtained by averaging the performance of all the five sets. In this study, we have also used jack-knife cross validation or Leave One Out Validation (LOOV) technique for evaluating performance of our models. In this technique, one sample is used for testing and remaining samples for training, this process is repeated in such a manner that each sample is used only once for testing.

### Support vector machine

We have used a highly successful machine learning classifier known as SVM for building prediction models. Therefore, we implemented SVM^light^ Version 6.02 package of SVM [27] and machine learning was carried out using various kernels (*e.g.* linear, polynomial, radial basis function and sigmoid tanh), where each input dot is converted into nonlinear kernel function. Here, we used RBF kernel of SVM at different parameter; g ∈ [10^-4^ - 10], c ∈ [1-15], j ∈ [1-5] for optimizing the SVM performance to get the best performance. SVM requires a set of fixed length of input features for training, thus necessitating a strategy for encapsulating the global information about proteins/peptides of variable length in a fixed length format. The fixed length format was obtained from protein/peptide sequences of variable length using amino acid composition, dipeptide composition and binary profile of pattern. After training, learned model can be used for the prediction of unknown examples.

### Amino acid composition

Peptide information can be encapsulated in a vector of 20 dimensions, using amino acid composition of the peptide. The amino acid composition is the fraction of each amino acid type within a peptide. The fractions of all 20 natural amino acids were calculated by using the following equation:

$$\mathrm{Comp}\left(i\right)=\frac{R_{i}}{N}\times 100$$

Where Comp (*i*) is the percent composition of amino acid (*i*); *R*_*i*_ is number of residues of type *i*, and *N* is the total number of residues in the peptide.

### Dipeptide composition

The dipeptide composition provides composition of pair of residues (*e.g.* Ala-Ala, Ala-Leu, *etc.*) present in peptide, and used to transform the variable length of peptides to fixed length feature vectors. It gives a fixed pattern length of 400 (20 × 20), and encapsulates information about the fraction of amino acids as well as their local order. It is calculated using following equation:

$$\begin{array}{l} \mathrm{Fraction}\;\mathrm{of}\;\mathrm{Dipeptide}\;\left(i\right)\\ \;=\frac{\mathrm{Total}\;\mathrm{number}\;\mathrm{of}\;\mathrm{Dipeptide}\;\left(i\right)}{\mathrm{Total}\;\mathrm{number}\;\mathrm{of}\;\mathrm{all}\;\mathrm{possible}\;\mathrm{dipeptides}} \end{array}$$

Where dipeptide (*i*) is one out of 400 dipeptides.

### Binary profile of patterns

Binary profiles were generated for each peptide, where each amino acid is represented by a vector of dimensions of 20 (*e.g.* Ala by 1,0,0,0,0,0,0,0,0,0,0,0,0,0,0,0,0,0,0,0) as described in supporting information (Additional file 1: Figure S1). A pattern of window length W was represented by a vector of dimensions 20 × W. We have created binary profile for first 5 and 10 residues from N-terminus, similarly for last 5 and 10 residues from C-terminus of peptides in all datasets. The binary profile has been used in a number of existing methods [28,29].

### Physicochemical properties

Physicochemical properties like amphipathicity, hydrophobicity, charge, length, *etc.* have been previously shown to be useful in the prediction of CPPs [20,22]. We have calculated these properties (amphipathicity, hydrophobicity, charge, molecular weight, length, isoelectric point, side chain bulk, steric bulk, net donated hydrogen bonds, and number of polar and non-polar residues) of amino acids to develop prediction models for CPPs. We have taken numerical values of these physicochemical properties from latest version of AA index database [30].

### Sequence logos

The sequence logos were generated using online WebLogo software [31]. The sequence logo gives the position specific frequency of amino acids in peptides. Each logo consists of stacks of symbols, one stack for each position in the sequence. The overall height of the stack indicates the sequence conservation at that position, while the height of symbols within the stack indicates the relative frequency of each amino acid at that position.

### MEME/MAST motifs

We have observed various common patterns/motifs in CPPs. In order to identify motifs in CPPs, we have used MEME/MAST program [32]. In the present study, meme-4.7.0 version was used. We got the number of motifs in CPPs using MEME, and these motifs have been used further to scan peptides for the presence of CPP specific motifs using program MAST. Hits obtained in the MAST output were used to calculate the efficacy and coverage of MEME/MAST method. E-value is very crucial in the MAST output, so we took this into account and calculated the efficacy of this method at different E-values (10-10^-7^).

### Hybrid approach

In hybrid approach, we have combined SVM output with motif information obtained by MEME/MAST for the better and biologically reliable prediction of CPPs. In this approach, for a query peptide, first SVM model is applied and it generates an SVM score. In parallel, the query peptide is searched against the CPP motifs, if any motif is found in the peptide; its SVM score is increased by a value of 5, so that in any case, it would be predicted as positive whatever is the original prediction.

### Performance measure

The performance of various models developed in this study was computed using threshold-dependent as well as threshold-independent parameters. In threshold dependent parameters we used sensitivity (Sn), specificity (Sp), overall accuracy (Ac) and Matthew’s correlation coefficient (MCC) using following equations.

$$\mathrm{Sensivity}=\frac{\mathrm{TP}}{\mathrm{TP}+\mathrm{FN}}\times 100$$

$$\mathrm{Specificity}=\frac{\mathrm{TN}}{\mathrm{TN}+\mathrm{FP}}\times 100$$

$$\mathrm{Accuracy}=\frac{\mathrm{TP}+\mathrm{TN}}{\mathrm{TP}+\mathrm{FP}+\mathrm{TN}+\mathrm{FN}}\times 100$$

$$\mathrm{MCC}=\frac{\left(\mathrm{TP}\;\text{x}\;\mathrm{TN}\right)-\left(\mathrm{FP}\;\text{x}\;\mathrm{FN}\right)}{\left(\mathrm{TP}+\mathrm{FP}\right)\;\left(\mathrm{TP}+\mathrm{FN}\right)\;\left(\mathrm{TN}+\mathrm{FP}\right)\;\left(\mathrm{TN}+\mathrm{FN}\right)}$$

Where TP and TN are correctly predicted positive and negative examples, respectively. Similarly, FP and FN are wrongly predicted positive and negative examples respectively.

We created ROC (Receiver Operating Characteristic) for all of the models in order to evaluate performance of models using threshold-independent parameters. ROC plots with area under curve (AUC) were created using ROCR statistical package available in R [33].

## Results

### Amino acid composition analysis of CPPs

**Figure 1:**
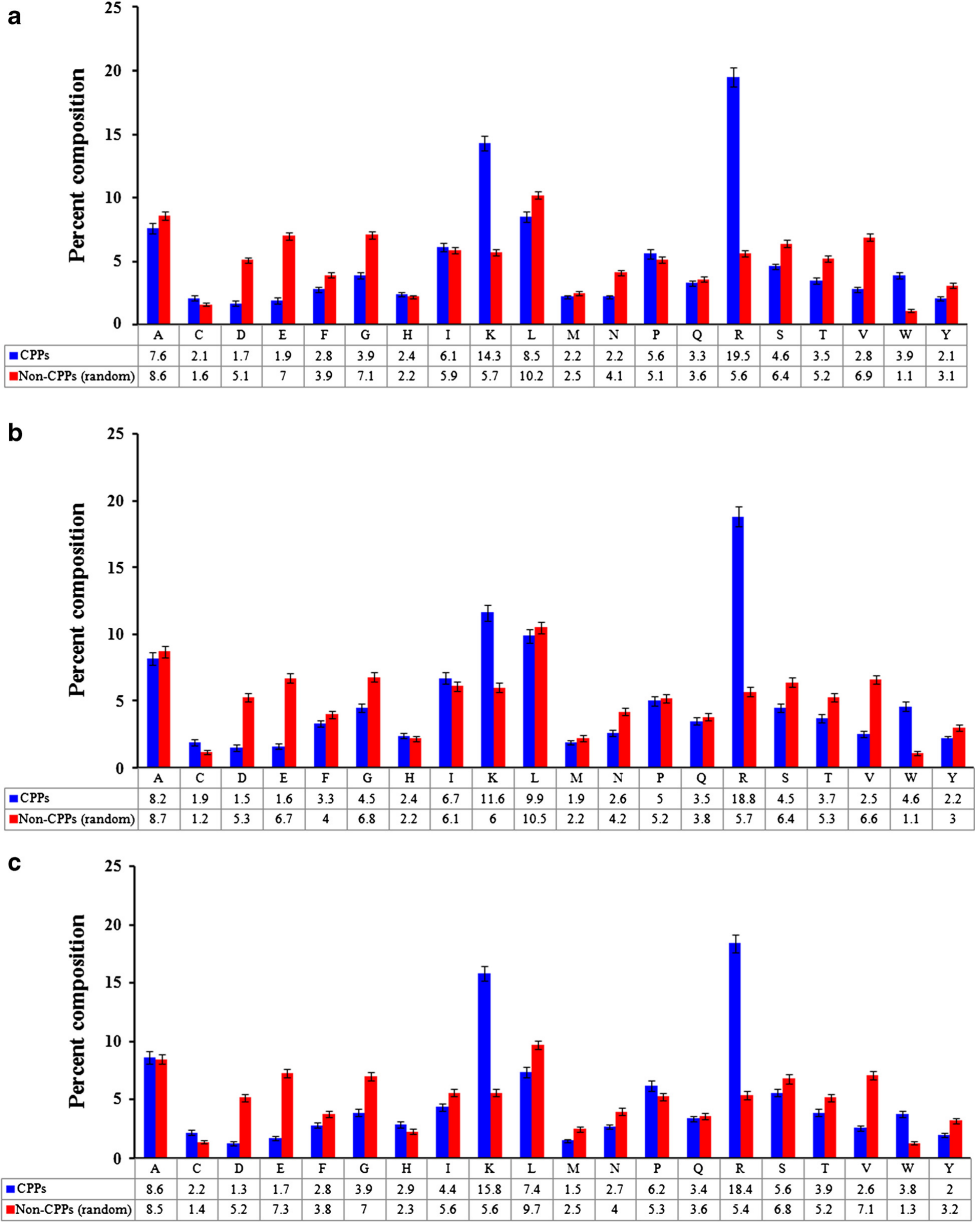
**Amino acid composition comparison.** Comparison of percent average amino acid composition of (**a**) whole peptides, (**b**) N-terminal residues, and (**c**) C-terminal residues between CPPs and non-CPPs.

In order to understand whether certain types of amino acids are dominated in CPPs, overall percent average composition of amino acids in CPPs and non-CPPs has been calculated and compared (Figure 1a). Analysis revealed that Arg, Lys, and Trp were significantly abundant in CPPs, while composition of Pro and Cys were slightly higher in CPPs than non-CPPs (Figure 1a). Next, we wanted to know whether certain types of residues are dominated at N- and C- terminus. To address this, we have computed percent average residue composition of both N- and C- termini (spilt amino acid composition). However, we did not observe significant difference in split amino acid composition from the overall residue composition in CPPs (Figure 1b and 1c).

### Residues preference in CPPs

**Figure 2:**
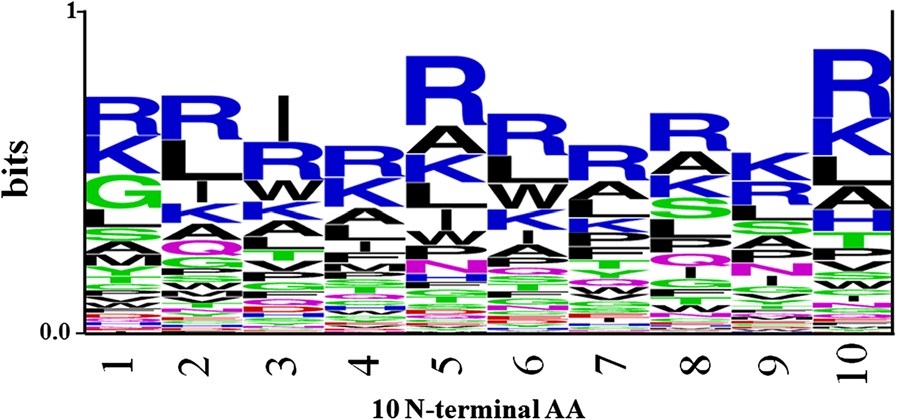
**Sequence logo of first ten residues (N-terminus) of CPPs.** The figure depicts the sequence logo of first ten residues (N-terminus) of CPPs, where size of residue is proportional to its propensity.

**Figure 3:**
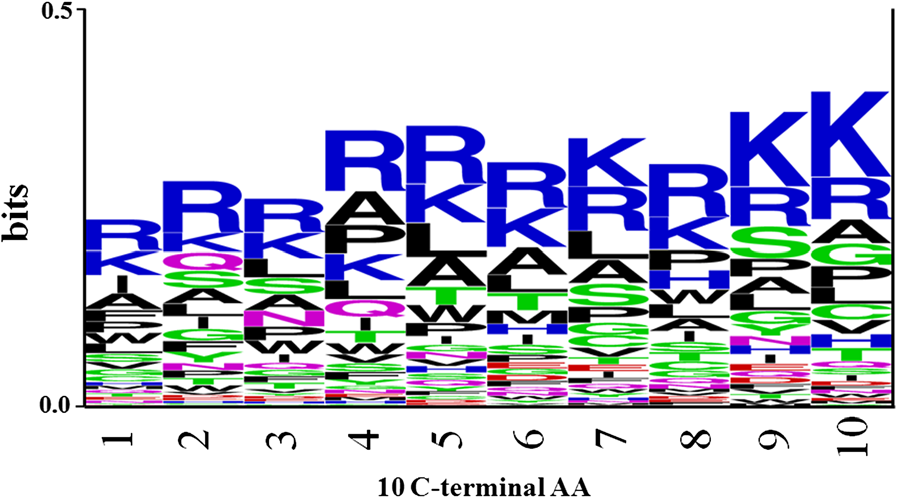
**Sequence logo of last ten residues (C-terminus) of CPPs.** The figure depicts the sequence logo of last ten residues (C-terminus) of CPPs, where size of residue is proportional to its propensity.

We next analyzed whether certain types of residues are preferred at specific positions in CPPs. To understand this, frequency of occurrence of all amino acids at both the termini was examined. It was observed that particular types of residues are preferred over others in CPPs at N- and C-terminus. In order to demonstrate residue preference at different position of CPPs, sequence logos [31] were generated. The sequence logos of 10 N-terminal and C-terminal residues of peptides are shown in Figure 2 and 3 respectively. It is clearly depicted in Figure 2 and 3 that basic residues (Arg and Lys) are preferred at most of the positions. However, certain residues like Leu, Ala, Ile, and Trp (at N-terminus) and Leu, Ser, and Pro (at C-terminus) are also preferred at various positions in CPPs.

### Amino acid composition-based SVM model

It has been shown in the past that amino acid composition can be used to classify the different classes of peptides and to develop prediction tools using machine-learning techniques [34,35]. In composition analysis, we have found that certain types of residues are preferred over the others in CPPs. Thus, it was possible to discriminate CPPs from non-CPPs based on amino acid composition. Therefore, we have developed an SVM model using amino acid composition as input feature. The performance of this model is summarized in Table 1. For CPPsite-1, we have found a maximum accuracy of 90.47% with MCC and ROC values 0.81 and 0.96, respectively. In case of CPPsite-2, we have achieved maximum accuracy of 90.37% with MCC and ROC values 0.81 and 0.96, respectively. For dataset CPPsite-3, we have achieved a maximum accuracy of 68.98% with MCC and ROC values 0.38 and 0.73, respectively. This indicates that performance of this model on CPPsite-3 dataset (which discriminates high and low efficient CPPs) is relatively poor.

**Table 1:** Performance of composition-based SVM method

**Dataset**	**Sensitivity**	**Specificity**	**Accuracy**	**MCC**	**ROC**
**CPPsite-1**	89.12	91.81	90.47	0.81	0.96
**CPPsite-2**	92.51	88.24	90.37	0.81	0.96
**CPPsite-3**	70.59	67.38	68.98	0.38	0.73

### Dipeptide composition-based SVM model

Since the dipeptide encapsulates the global information of the amino acid fraction and the local order of amino acids, it is a better feature as compared to amino acid composition alone. Dipeptide composition has been used in earlier studies to differentiate two different types of proteins and peptides [36]. Thus, we have developed an SVM model based on dipeptide composition. This model performed more or less similar to composition–based model. Results are shown in Table 2. In case of CPPsite-1, we achieved a maximum accuracy of 90.04% with MCC and ROC values of 0.80 and 0.95, respectively. For CPPsite-2, maximum accuracy achieved was 92.78% with MCC and ROC values of 0.86 and 0.97, respectively. For CPPsite-3, maximum accuracy was 67.11% with MCC and ROC values of 0.34 and 0.71, respectively.

**Table 2:** Performance of dipeptide-based SVM method

**Dataset**	**Sensitivity**	**Specificity**	**Accuracy**	**MCC**	**ROC**
**CPPsite-1**	88.14	91.95	90.04	0.80	0.95
**CPPsite-2**	90.91	94.65	92.78	0.86	0.97
**CPPsite-3**	72.73	61.50	67.11	0.34	0.71

### Binary profile-based SVM model

In preliminary analysis, certain residues (Ala, Pro, Leu, Ile, Trp, Ser) along with Arg and Lys are also preferred at various positions at N- and C-terminus. To incorporate this position specific information in the model, we have generated binary profile patterns of peptides. In binary pattern, a vector of dimension 20 represents a residue, and for n residues the input vector of dimension is (20 × n). We have used the following three approaches:

#### N-terminal approach

In this approach, we have extracted 5 and 10 N-terminus residues from each peptide in all three datasets, and generated binary profile of dimension 5×20 and 10×20 respectively. These profiles were then used to develop SVM model. Comparisons of performances of binary-based SVM models are shown in Table 3. Model developed on CPPsite-2 dataset performed better than models developed on other two datasets.

**Table 3:** Performance of binary profile-based SVM method

**Method**	**CPPsite-1 dataset**					**CPPsite-2 dataset**					**CPPsite-3 dataset**				
	**Sn**	**Sp**	**AC**	**MCC**	**ROC**	**Sn**	**Sp**	**AC**	**MCC**	**ROC**	**Sn**	**Sp**	**AC**	**MCC**	**ROC**
**N5**	80.08	85.73	82.91	0.66	0.89	86.63	87.17	86.90	0.74	0.90	62.03	65.78	63.90	0.28	0.64
**C5**	84.60	82.20	83.40	0.67	0.91	91.44	82.35	86.90	0.74	0.95	64.17	67.38	65.78	0.32	0.66
**N5-C5**	83.19	88.98	86.09	0.72	0.96	91.98	82.35	87.17	0.75	0.95	66.84	66.84	66.84	0.34	0.69
**N10**	83.95	86.19	85.03	0.70	0.91	89.44	90.34	89.87	0.80	0.95	66.67	63.27	65.05	0.30	0.65
**C10**	86.55	83.22	84.95	0.70	0.93	87.04	91.10	88.96	0.78	0.95	66.05	61.90	64.08	0.28	0.68
**N10-C10**	90.60	86.89	88.81	0.78	0.95	93.21	93.84	93.51	0.87	0.96	66.67	64.63	65.70	0.31	0.68

*Sn*: sensitivity, *Sp*: specificity, *AC*: accuracy.

#### C-terminal approach

We have used the same strategy for the C-terminus as used for the N-terminus. The performance of binary-based SVM model using 5 and 10 C-terminal residues was almost similar to N-terminal approach (Table 3).

#### N + C-terminal approach

In order to check, if using the N- and C-termini of the peptides together will enhance the accuracy of the method or not, we developed an N + C-terminus based approach. In this approach, we have developed two datasets, named N5-C5 and N10-C10. First 5 residues from the N-terminal were joined with 5 residues from C-terminal in N5-C5 dataset. Similarly in N10-C10, first 10 residues from N-terminal were joined with last 10 residues from C-terminal. The comparative performances of binary-based SVM model using N + C terminal residues are shown in Table 3. For CPPsite-1, CPPsite-2 and CPPsite-3 datasets, maximum accuracy of 88.81%, 93.51% and 66.84% was achieved respectively. This model performed better in case of CPPsite-2 dataset, than the models based on above two approaches.

### Physicochemical properties-based SVM model

For each dataset, we have calculated a set of physicochemical properties (described in material and methods) of each peptide, which were previously shown to be useful for prediction of CPPs [20]. SVM model using these physicochemical properties has been developed. Performance of this model was similar to composition-based model. Results are summarized in Table 4. For CPPsite-1, we have achieved maximum accuracy of 90.75% with MCC and ROC values of 0.82 and 0.95, respectively. For CPPsite-2, maximum accuracy of 90.91% with MCC and ROC values of 0.82 and 0.95 respectively, was achieved. For CPPsite-3, maximum accuracy of 68.72% with MCC and ROC values, of 0.32 and 0.70 respectively, was achieved.

**Table 4:** Performance of physicochemical properties-based SVM method

**Dataset**	**Sensitivity**	**Specificity**	**Accuracy**	**MCC**	**ROC**
**CPPsite-1**	91.24	90.25	90.75	0.82	0.95
**CPPsite-2**	91.98	89.84	90.91	0.82	0.95
**CPPsite-3**	73.80	63.64	68.72	0.32	0.70

### Cross-validation techniques

We have evaluated our models using five-fold cross validation and LOOCV techniques. As shown in supporting information (Additional file 1: Tables S1-S4), performance of models was nearly same when evaluated using LOOCV or using five-fold cross-validation technique. Therefore, for the further studies on CPPs prediction, we have used five-fold cross validation only, because it is less expensive in terms of time and computer usage as compared to the LOOCV.

### MEME/MAST motif based method

In the previous studies, motif information has been used for the prediction of other biological problems *e.g.* prediction of sub-cellular localization of proteins [37]. We have observed various motifs in CPP datasets. These motifs were fished out using MEME software with E-value of 10. Subsequently, this motif information has been used for the prediction of CPPs. We have repeated the motif-based method at different E-values form 10 to 10^-7^ for each dataset. Results of all the three datasets are presented in Table 5. Here, it should be noticed that probability of correct prediction is satisfying, but on the other hand, percent coverage is not recommendable in all the three datasets as shown in Table 5.

**Table 5:** Performance of MEME/MAST-based SVM method

**E-value**	**CPPsite-1**		**CPPsite-2**		**CPPsite-3**	
	**PCP**	**% Coverage**	**PCP**	**% Coverage**	**PCP**	**% Coverage**
**10**	0.50	81.17	0.48	79.88	0.54	79.88
**1**	0.50	74.40	0.48	74.71	0.56	74.71
**0.1**	0.48	63.10	0.50	69.54	0.60	69.54
**0.01**	0.5	54.97	0.53	62.64	0.63	62.64
**1E-02**	0.56	50	0.57	56.32	0.64	56.32
**1E-04**	0.64	45.03	0.62	52.87	0.65	52.87
**1E-05**	0.74	42.92	0.70	51.14	0.66	51.14
**1E-06**	0.83	39.46	0.83	48.28	0.66	48.28
**1E-07**	0.90	36.45	0.88	45.98	0.68	45.98

*PCP*: Percentage of correct prediction.

### Hybrid prediction model

As we noticed, that MEME/MAST method has excellent ability to predict CPPs, but with very little coverage (Table 5). Therefore, we have developed a hybrid method by combining MEME/MAST method with the binary pattern profile-based SVM model in order to take the advantage of accuracy of MEME/MAST method. Hybrid model achieved maximum accuracies (at E-value 10) of 92.85%, 97.40% and 78.96% for CPPsite-1, CPPsite-2 and CPPsite-3 datasets respectively. Results of hybrid approach are shown in Table 6.

**Table 6:** Performance of hybrid method

**E-Value**	**CPPsite-1 dataset**					**CPPsite-2 dataset**					**CPPsite-3 dataset**				
	**Sn**	**Sp**	**AC**	**MCC**	**ROC**	**Sn**	**Sp**	**AC**	**MCC**	**ROC**	**Sn**	**Sp**	**AC**	**MCC**	**ROC**
**10**	91.90	93.88	92.85	0.86	0.97	98.15	96.58	97.40	0.95	0.99	80.86	76.87	78.96	0.58	0.86
**1**	91.41	93.88	92.60	0.85	0.97	96.91	96.58	96.75	0.93	0.99	79.01	76.87	77.99	0.56	0.84
**0.1**	91.25	93.88	92.51	0.85	0.97	95.68	96.58	96.10	0.92	0.99	76.54	76.87	76.70	0.53	0.83
**0.01**	90.76	93.88	92.26	0.85	0.97	95.06	96.58	95.78	0.92	0.99	74.07	76.87	75.40	0.51	0.81
**1E-02**	89.63	93.88	91.67	0.83	0.97	94.44	96.58	95.45	0.91	0.98	71.60	76.87	74.11	0.48	0.79
**1E-04**	88.65	93.88	91.17	0.83	0.97	94.44	96.58	95.45	0.91	0.98	53.09	76.87	64.40	0.31	0.68
**1E-05**	88.17	93.88	90.92	0.82	0.96	94.44	96.58	95.45	0.91	0.98	53.09	76.87	64.40	0.31	0.68
**1E-06**	88.01	93.88	92.83	0.82	0.96	94.44	96.58	95.45	0.91	0.98	53.09	76.87	64.40	0.31	0.68
**1E-07**	87.52	93.88	90.58	0.81	0.96	94.44	96.58	95.45	0.91	0.98	70.59	67.38	68.98	0.38	0.73

*Sn*: sensitivity, *Sp*: specificity, *AC*: accuracy.

### ROC plot

**Figure 4:**
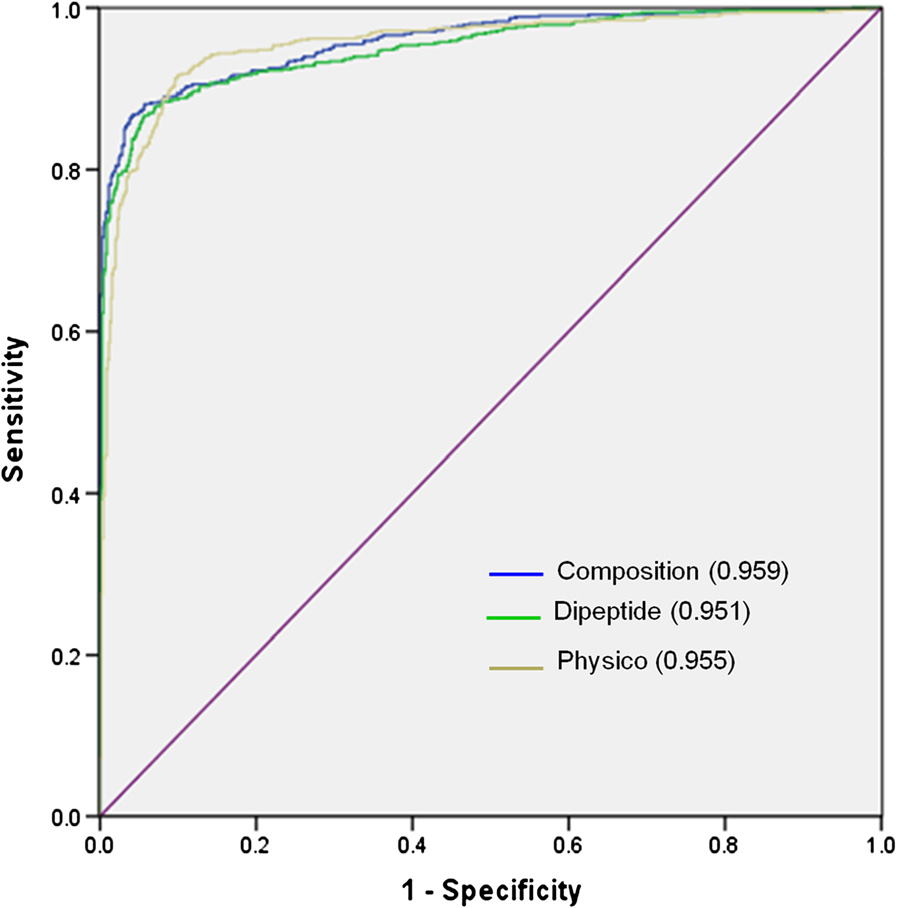
**The performance of SVM models developed using composition, dipeptide and physicochemical property profile on CPPsite-1 dataset (where 1-specificity represents the false positive rate and value in bracket shows area under curve).**

**Figure 5:**
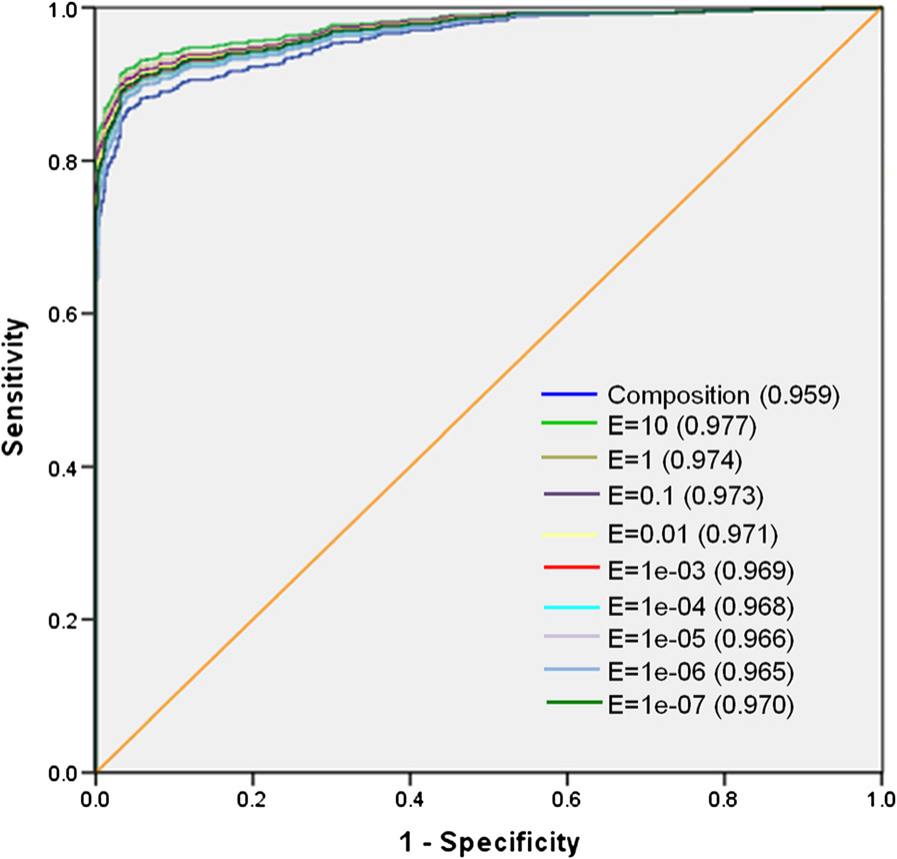
**The performance of SVM models developed using composition and hybrid models on CPPsite-1 dataset (where 1-specificity represents the false positive rate and value in bracket shows area under curve).**

In order to have a threshold-independent evaluation of our models, we have generated ROC curve for all the models. ROCR statistical package was used for creating ROC plots with area under curves (AUC). As shown in Figure 4, composition-based method performed well over the other methods. However, when we compared composition-based method with the hybrid method, hybrid method performed well as compared to the composition-based method at all the E-values (Figure 5).

### Comparison with existing methods

In order to validate our method, performance of binary-based method was evaluated on independent dataset and we achieved 81.31% accuracy with 0.63 MCC. In addition, we developed and evaluated our models on benchmark datasets. A comparison of previously published prediction methods with our approach is shown in Table 7 and supporting information (Additional file 1: Table S5). These results clearly demonstrate that hybrid model is more accurate than previous methods and may complement the existing methods.

**Table 7:** Comparison with previous methods

**Benchmark datasets**	**Previous accuracy**	**Accuracy of models**		
		**Composition based model**	**Dipeptide based model**	**Hybrid model**
**Sanders-2011a**	95.94	96.40	98.65	97.75
**Sanders-2011b**	75.86	82.07	83.45	83.45
**Sanders-2011c**	88.73	88.74	89.64	90.09
**Dobchev-2010**	83.16	81.63	81.63	83.33
**Hansen-2008**	67.44	78.82	83.53	80.00
**Hallbrink-2005**	77.27	92.75	95.65	97.06

### Implementation and designing of CPPs

**Figure 6:**
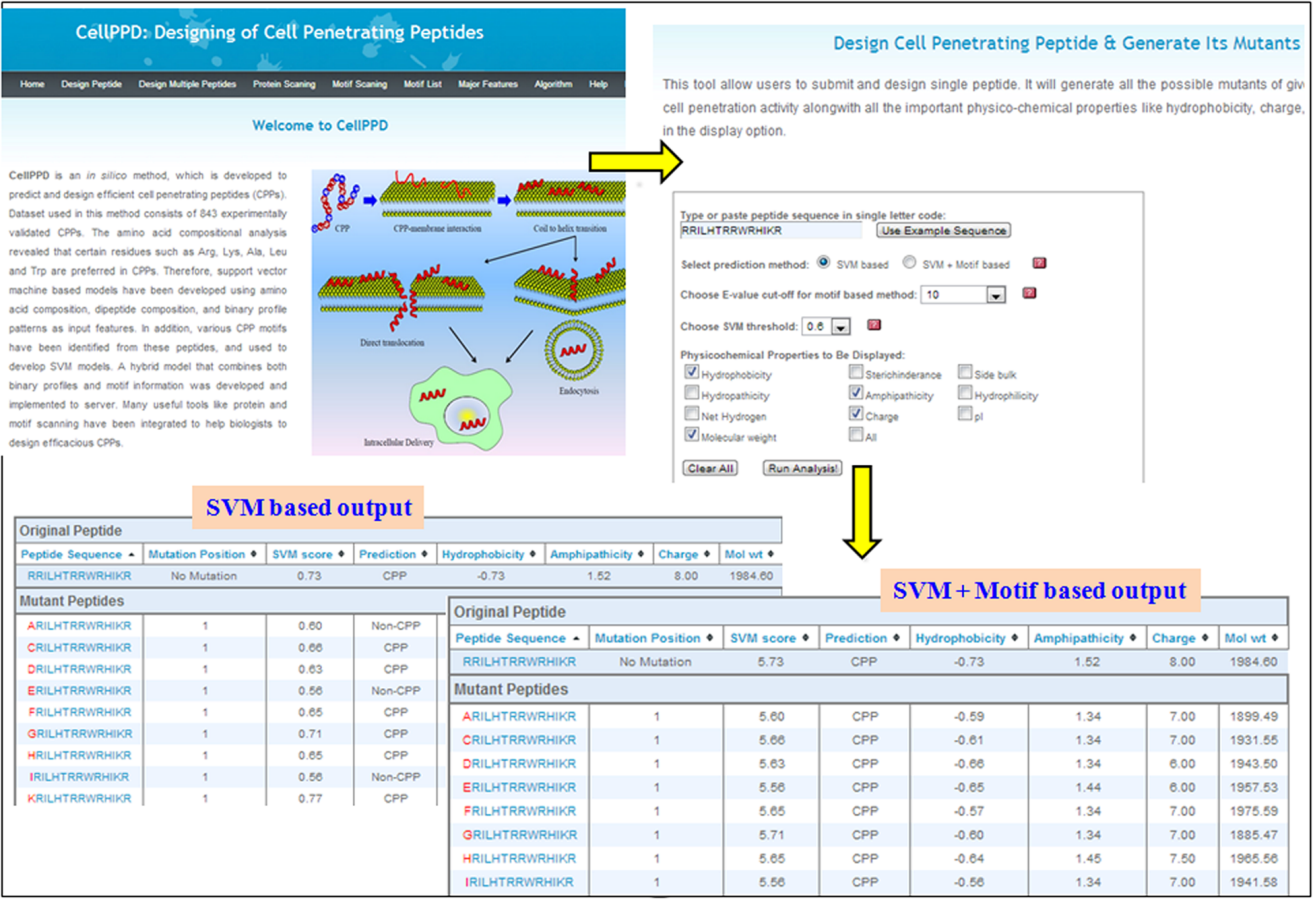
Schematic presentation of CellPPD webserver with an example of SVM based prediction results.

Currently, no web service is available for the prediction of CPPs till date. Thus, in order to serve scientific community, we have implemented our best methods (binary N10-C10 and hybrid) in a user-friendly web server ‘CellPPD’ with many other useful tools for the users (Figure 6). CellPPD web server not only provides facility to predict peptides as CPPs or non-CPPs, but also it offers opportunity to design analogues with better cell penetrating abilities. The detailed information related to designing of CPP analogues has been provided in supporting information (Additional file 2). User may submit the peptide sequence (no FASTA format required) in single letter code, and server will generate all the possible mutants of given peptide with single mutation in each mutant (depicted in red color). For each mutant peptide, server will give an SVM score and prediction status CPP or non-CPP according to the threshold cut-off chosen by the user. As this server allows users to select a threshold, we suggest the users to select higher value if they are interested in high specificity (high confidence). Therefore, this feature will be very helpful for user in designing highly effective CPP analogues. In addition, server also calculates important physicochemical properties in an aesthetic table format (Figure 6). In the same table, original peptide will also be displayed and sorting option has been provided, which can be used to sort the peptide analogues based on desired properties and eventually to select the best peptide analogue. There is a provision to submit and design multiple peptides at a time. For this, user has to submit multiple sequences in FASTA format. Another informative tool is the scanning of protein for the detection of putative CPPs. Here, user may submit the protein sequence, and server will generate overlapping peptides of window length selected by the user, where all the peptides will be clickable. This tool can help users to dig out a protein sequence for possible CPPs. Graphical representation of results is an interesting feature providing an estimate of total CPPs containing regions in the protein. Motif scanning is another handy tool for the user to find CPP motifs in a protein sequence. We have also provided a list of 120 CPP motifs present in our dataset of CPPs. In addition, few examples (prediction test on well-known CPPs and their non-penetrating non-CPP analogues) have been incorporated in supporting information (Additional file 2) for accuracy comparison of our method. CellPPD is freely accessible at http://crdd.osdd.net/raghava/cellppd.

## Discussion

Due to huge therapeutic applications of CPPs, especially in drug delivery, identification of novel and highly efficient CPPs is need of the hour. However, identification of highly efficient CPPs is a very tedious task for biologists. One has to scan the whole protein in overlapping window patterns, and every peptide has to be tested for the possible cell penetrating activity, which is a very laborious and time consuming cycle. A computational method, which can determine whether a peptide sequence can be a CPP or not, would definitely help biologists for rapid screening of CPPs before synthesis and thus, accelerate the CPP-based research. The development of an *in silico* method for CPP prediction is very challenging due to three major reasons; (i) CPPs have lot of variation in size (5 – 30 amino acids), and machine learning software need fixed length patterns as input to develop model, (ii) experimentally proven non-CPPs (negative dataset) are not reported in literature, which are very important for developing the *in silico* method, and (iii) other major problem in CPP prediction is the lack of dataset of peptides (CPPs and non-CPPs) tested in similar experimental conditions (*e.g.* concentrations, incubation time, cell lines, type of cargoes, *etc.*). In most of the CPP-based research, uptake of peptides has been tested on different cell lines with different experimental conditions. It could be possible that few non-penetrating analogues of previously known CPPs may act as CPP when evaluated on alternative cell lines or in different experimental conditions. Sanders *et al.* have also observed a similar observation, where a previously known non-CPP found to have some penetrating properties when tested on different cell lines (*i.e.* avian cell line) [22]. Therefore, for the better and more accurate prediction, larger dataset of CPPs and non-CPPs tested in number of cell lines with similar experimental conditions are required. However, in the past, few attempts have been made to predict CPPs [19-22], but all these methods used very small dataset and none of these has provided web service. In the last decade, a large amount of data on the use of CPPs as delivery agents has accumulated and this enormous growth of CPP data motivated us to develop an *in silico* method on a larger dataset of 708 experimentally validated CPPs. In order to develop a robust computational method, which can discriminate CPPs from non-CPPs with higher accuracy, we have developed SVM models on three datasets (CPPsite-1, CPPsite-2 and CPPsite-3) using many features like amino acid composition, dipeptide composition, binary pattern of profile and CPP motifs.

Performances of SVM models developed on dataset CPPsite-1 and CPPsite-2 were significantly better than models developed on CPPsite-3 dataset. This is due the fact that in CPPsite-3, both positive and negative examples are CPPs; the only difference is that positive examples consist of CPPs with high uptake efficiency, while negative examples consist of CPPs with low uptake efficiency. Since peptides in both the classes are CPPs and contain similar properties including amino acid composition (Additional file 1: Figure S2), they are difficult to discriminate.

SVM models using amino acid and dipeptide composition as input features performed reasonably good and achieved more or less similar accuracy. Recently, Sanders *et al.* (2011) published a method, in which they have used amino acid compositions and 41 other biochemical properties, including amino acid frequency, length, hydrophobicity, *etc.* as an input feature to develop SVM model. We have shown that amino acid composition alone can predict CPP with better accuracy (Table 7). The dipeptide-based model achieved greater accuracy (98%) for Sanders-2011a dataset, while the increase in accuracy (95.94% to 96.40%) for whole amino acid composition-based model for Sanders-2011a dataset is negligible and could be due to the random sampling of negative examples. One of the limitations in composition-based model is that it only computes the overall number of residues in peptides and loses the amino acid order information, which is equally important. It is well known that the peptide’s function is strongly related to its sequence order. Evidence suggests that conformation of CPPs plays a crucial role in membrane interaction and insertion [38]. It has been shown that CPP with helical conformation can penetrate membrane more effectively than the peptides with other conformations [38]. Many amphipathic CPPs adopt helical conformation in which all the polar residues grouped at one face and the nonpolar residues to the opposite face of the helix. This amphipathic helical distribution can also be associated to specific amino acids and with a particular order. In addition, preliminary analysis (Figures 2 and 3) has also shown that certain residues are preferred at specific positions in CPPs. Therefore, in order to include this information, we have developed SVM models based on binary profile of patterns, which incorporates information of both composition and amino acid order. In many previous studies, binary profiles patterns-based SVM model performed better than composition-based model [25,26]. In this study also, N10-C10 binary profile-based SVM model achieved maximum accuracy (93.51%) in CPPsite-2 dataset.

In addition, we have also developed motif-based method using MEME/MAST, where MEME is used to discover motifs and MAST is used to search these motifs in CPPs. We conducted our study keeping in mind that the CPPs might share some patterns/motifs. This approach has been used successfully in the past to differentiate two different classes of peptides [37]. In the present study also, the model developed on motif-based approach has predicted CPPs with reasonable accuracy. Finally, in order to improve performance of the model, a hybrid model using both binary profile patterns and motif information was developed. Motif information has further increased the accuracy of CPP prediction. We also compared our method with existing methods on benchmark datasets. The performance of our method was better than existing methods. Furthermore, in order to help biologists, we have implemented our best models in a user-friendly web server CellPPD.

## Conclusions

There is a rapid growth in the field of CPP research in response to the demand for novel drug delivery systems. CellPPD is one such efficient method that can predict highly efficient CPPs and help to find newer CPP analogues more speedily and conveniently. We hope that establishment of such method will speed up the pace of identifying improved and efficacious CPPs in future.

## Abbreviations

CPP: Cell penetrating peptides; PTD: Protein transduction domain; ANN: Artificial neural network; QSAR: Quantitative structure activity relationship; SVM: Support vector machine; LOOCV: Leave one out cross-validation; AUC: Area under curve; ROC: Receiver operating characteristic.

## Competing interests

The authors declare that they have no competing interests.

## Authors’ contributions

AG collected the data and created the datasets. KC, RK and AS developed computer programs, implemented SVM. KC, RK and AS created the back end server. KC, RK, AG, PK and AT developed the front end user interface. AG and RK wrote the manuscript. GPSR conceived and coordinated the project, helped in the interpretation of data, refined the drafted manuscript and gave overall supervision to the project. All of the authors read and approved the final manuscript.

## Supplementary Material

Additional file 1: Figure S1 Generation of binary profile of pattern. **Figure S2.** Percent average amino acid composition of peptides in CPPsite-2 and CPPsite-3 datasets. **Table S1.** Performance of composition-based SVM method. **Table S2.** Performance of dipeptide-based SVM method. **Table S3.** Performance of physicochemical-based SVM method. **Table S4.** Performance of binary profile-based SVM method. **Table S5.** Performance on benchmark datasets. Click here for file

Additional file 2 **Designing of CPPs and case studies.** Describes the utility of CellPPD webserver in designing better cell penetrating analogues and explains the accuracy comparison of CellPPD using few examples (case studies). Click here for file
