# Supplementary material for: In silico approaches for designing highly effective cell penetrating peptides
Source: J Transl Med. 2013 Mar 22;11:74. doi: 10.1186/1479-5876-11-74 (PMC3615965; doi:10.1186/1479-5876-11-74)
Supplement: Additional file 1: Figure S1 — Generation of binary profile of pattern. Figure S2. Percent average amino acid composition of peptides in CPPsite-2 and CPPsite-3 datasets. Table S1. Performance of composition-based SVM method. Table S2. Performance of dipeptide-based SVM method. Table S3. Performance of physicochemical-based SVM method. Table S4. Performance of binary profile-based SVM method. Table S5. Performance on benchmark datasets. [file 1479-5876-11-74-S1.doc]

**Additional file 1**

**­­­­­­­*In silico* approaches for designing highly effective cell penetrating peptides**

Ankur Gautam, Kumardeep Chaudhary, Rahul Kumar, Arun Sharma, Pallavi Kapoor, Atul Tyagi, Open Source Drug Discovery Consortium, Gajendra P.S. Raghava


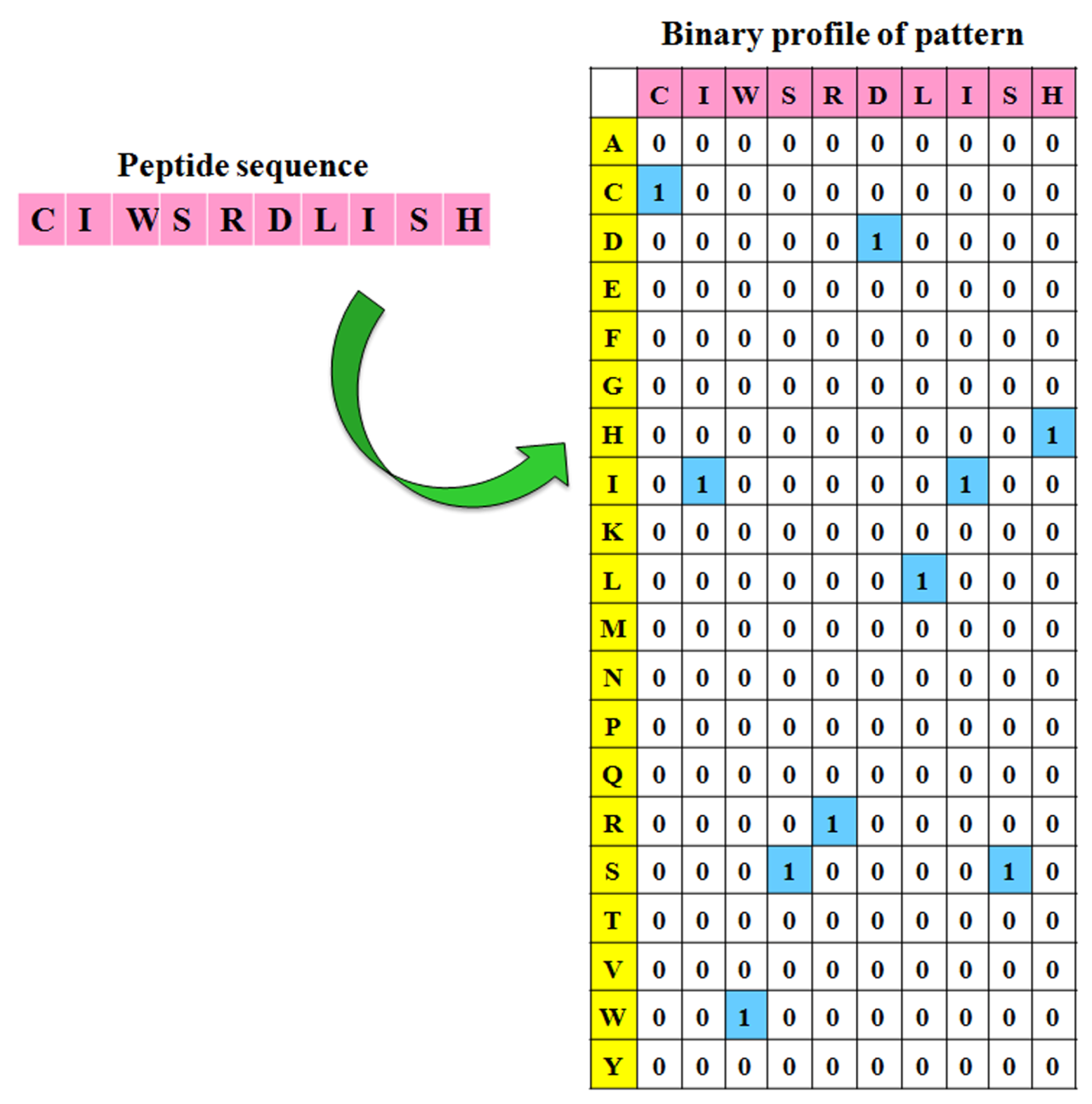


**Figure S1:** Generation of binary profile of pattern.


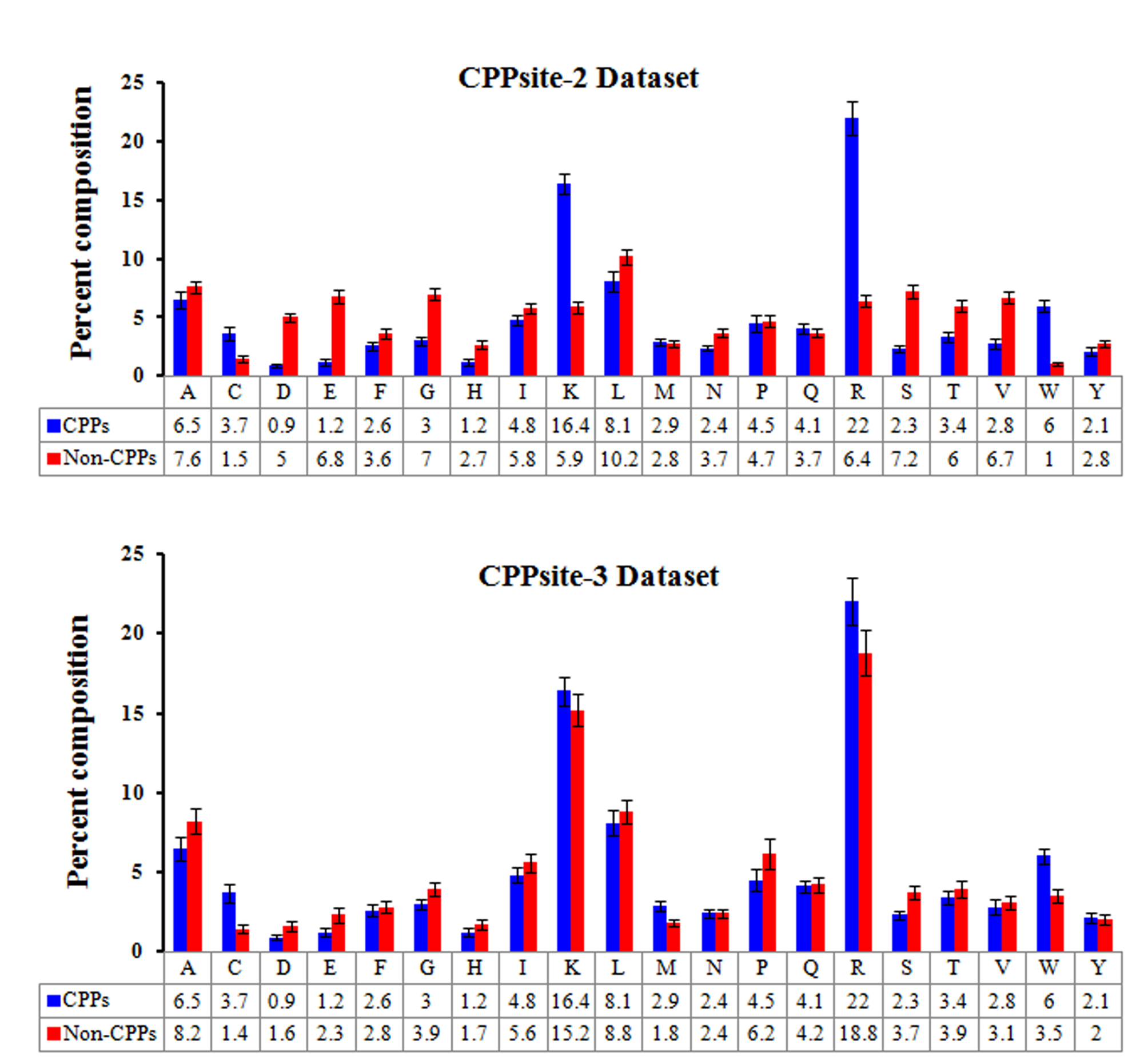


**Figure S2:** Percent average amino acid composition of peptides in CPPsite-2 and CPPsite-3 datasets.

**Table S1: Performance of composition-based SVM method.**

| **Dataset** | **Sensitivity** | **Specificity** | **Accuracy** | **MCC** | **ROC** |
| --- | --- | --- | --- | --- | --- |
| **CPPsite-1** | 90.11 | 92.09 | 91.10 | 0.82 | 0.96 |
| **CPPsite-2** | 90.37 | 87.17 | 88.77 | 0.78 | 0.96 |
| **CPPsite-3** | 66.84 | 65.24 | 66.04 | 0.32 | 0.70 |

**Table S2: Performance of dipeptide-based SVM method.**

| **Dataset** | **Sensitivity** | **Specificity** | **Accuracy** | **MCC** | **ROC** |
| --- | --- | --- | --- | --- | --- |
| **CPPsite-1** | 89.55 | 89.27 | 89.41 | 0.79 | 0.95 |
| **CPPsite-2** | 93.05 | 91.98 | 92.51 | 0.85 | 0.97 |
| **CPPsite-3** | 66.84 | 58.29 | 62.57 | 0.25 | 0.66 |

**Table S3: Performance of physicochemical-based SVM method.**

| **Dataset** | **Sensitivity** | **Specificity** | **Accuracy** | **MCC** | **ROC** |
| --- | --- | --- | --- | --- | --- |
| **CPPsite-1** | 91.24 | 90.25 | 90.75 | 0.82 | 0.95 |
| **CPPsite-2** | 92.51 | 91.44 | 91.98 | 0.84 | 0.96 |
| **CPPsite-3** | 66.31 | 63.64 | 64.97 | 0.30 | 0.69 |

**Table S4: Performance of binary-based SVM method.**

| **Method** | **CPPsite-1 dataset** | | | | |  | **CPPsite-2 dataset** | | | | |  | **CPPsite-3 dataset** | | | | |
| --- | --- | --- | --- | --- | --- | --- | --- | --- | --- | --- | --- | --- | --- | --- | --- | --- | --- |
|  | **Sn** | **Sp** | **AC** | **MCC** | **ROC** |  | **Sn** | **Sp** | **AC** | **MCC** | **ROC** |  | **Sn** | **Sp** | **AC** | **MCC** | **ROC** |
| **N5** | 79.38 | 85.45 | 82.42 | 0.65 | 0.89 |  | 85.56 | 85.03 | 85.29 | 0.71 | 0.89 |  | 62.57 | 59.36 | 60.96 | 0.22 | 0.60 |
| **C5** | 84.89 | 83.62 | 84.25 | 0.69 | 0.91 |  | 89.30 | 83.96 | 86.63 | 0.73 | 0.94 |  | 65.78 | 62.57 | 64.17 | 0.28 | 0.63 |
| **N5-C5** | 86.02 | 87.01 | 86.51 | 0.73 | 0.93 |  | 88.77 | 87.70 | 88.24 | 0.76 | 0.95 |  | 67.91 | 62.03 | 64.97 | 0.30 | 0.66 |
| **N10** | 85.58 | 85.14 | 85.37 | 0.71 | 0.91 |  | 89.44 | 87.59 | 88.56 | 0.77 | 0.95 |  | 62.96 | 61.22 | 62.14 | 0.24 | 0.63 |
| **C10** | 86.71 | 84.09 | 85.45 | 0.71 | 0.93 |  | 88.89 | 88.36 | 88.64 | 0.77 | 0.95 |  | 63.58 | 63.95 | 63.75 | 0.27 | 0.66 |
| **N10-C10** | 90.44 | 89.86 | 90.16 | 0.80 | 0.95 |  | 91.98 | 96.58 | 94.16 | 0.88 | 0.97 |  | 64.20 | 64.63 | 64.40 | 0.29 | 0.66 |

**Table S5: Performance on benchmark datasets.**

| **Benchmark Dataset** | **Composition based model** | | | |  | | **Dipeptide based model** | | | | | |  | | **Hybrid model** | | | | | | |
| --- | --- | --- | --- | --- | --- | --- | --- | --- | --- | --- | --- | --- | --- | --- | --- | --- | --- | --- | --- | --- | --- |
|  | **Sn** | **Sp** | **AC** | **MCC** | | **ROC** | |  | **Sn** | **Sp** | **AC** | **MCC** | | **ROC** | |  | **Sn** | **Sp** | **AC** | **MCC** | **ROC** |
| **Sanders-2011a** | 95.50 | 97.30 | 96.40 | 0.93 | | 0.99 | |  | 98.20 | 99.10 | 98.65 | 0.97 | | 0.99 | |  | 98.20 | 97.30 | 97.75 | 0.95 | 0.99 |
| **Sanders-2011b** | 96.40 | 35.29 | 82.07 | 0.43 | | 0.72 | |  | 90.09 | 61.76 | 83.45 | 0.53 | | 0.75 | |  | 98.20 | 35.29 | 83.45 | 0.48 | 0.90 |
| **Sanders-2011c** | 89.19 | 88.29 | 88.74 | 0.77 | | 0.91 | |  | 91.89 | 87.39 | 89.64 | 0.79 | | 0.95 | |  | 96.40 | 83.78 | 90.09 | 0.81 | 0.96 |
| **Dobchev-2010** | 98.65 | 25.00 | 80.61 | 0.39 | | 0.62 | |  | 97.30 | 33.33 | 81.63 | 0.44 | | 0.71 | |  | 95.83 | 33.33 | 80.21 | 0.40 | 0.85 |
| **Hansen-2008** | 89.39 | 42.11 | 78.82 | 0.34 | | 0.67 | |  | 98.48 | 31.58 | 83.53 | 0.46 | | 0.55 | |  | 95.08 | 31.58 | 80.00 | 0.36 | 0.85 |
| **Hallbrink-2005** | 98.11 | 75.00 | 92.75 | 0.79 | | 0.90 | |  | 96.23 | 93.75 | 95.65 | 0.88 | | 0.98 | |  | 98.08 | 93.75 | 97.06 | 0.92 | 0.98 |
