# Supplementary material for: In silico approaches for designing highly effective cell penetrating peptides
Source: J Transl Med. 2013 Mar 22;11:74. doi: 10.1186/1479-5876-11-74 (PMC3615965; doi:10.1186/1479-5876-11-74)
Supplement: Additional file 2 — Designing of CPPs and case studies. Describes the utility of CellPPD webserver in designing better cell penetrating analogues and explains the accuracy comparison of CellPPD using few examples (case studies). [file 1479-5876-11-74-S2.doc]

**Additional file 2**

**­­­­­­­*In silico* approaches for designing highly effective cell penetrating peptides**

Ankur Gautam, Kumardeep Chaudhary, Rahul Kumar, Arun Sharma, Pallavi Kapoor, Atul Tyagi, Open Source Drug Discovery Consortium, Gajendra P.S. Raghava

**Designing of cell penetrating peptide analogues using CellPPD**

CellPPD web server not only provides facility to predict peptides as CPP or non-CPP, but also it offers opportunity to design analogues with better cell penetrating abilities. Besides prediction of a given peptide, CellPPD also generates all possible single substitution mutants of original peptide with their SVM scores and prediction status. Along with this, server also calculates important physicochemical properties (*e.g.* hydrophobicity, amphipathicity, charge pI, *etc*.) in an aesthetic tabular format with sorting option. This feature is helpful for user to select better analogues based on desired physicochemical properties, as many peptide analogues may have higher SVM score or better-desired properties than the original peptide. In addition, user can further generates all possible mutants (2nd round) of their selected analogue if they wish to, and may get the even better peptide analogues with higher cell penetrating abilities (based on SVM score). This cycle (called CPP designing cycle, Figure 1) can be run until the peptide analogue with desired properties (cell penetrating and physicochemical) is obtained. One example is given below to explain designing of CPP analouges using CellPPD.


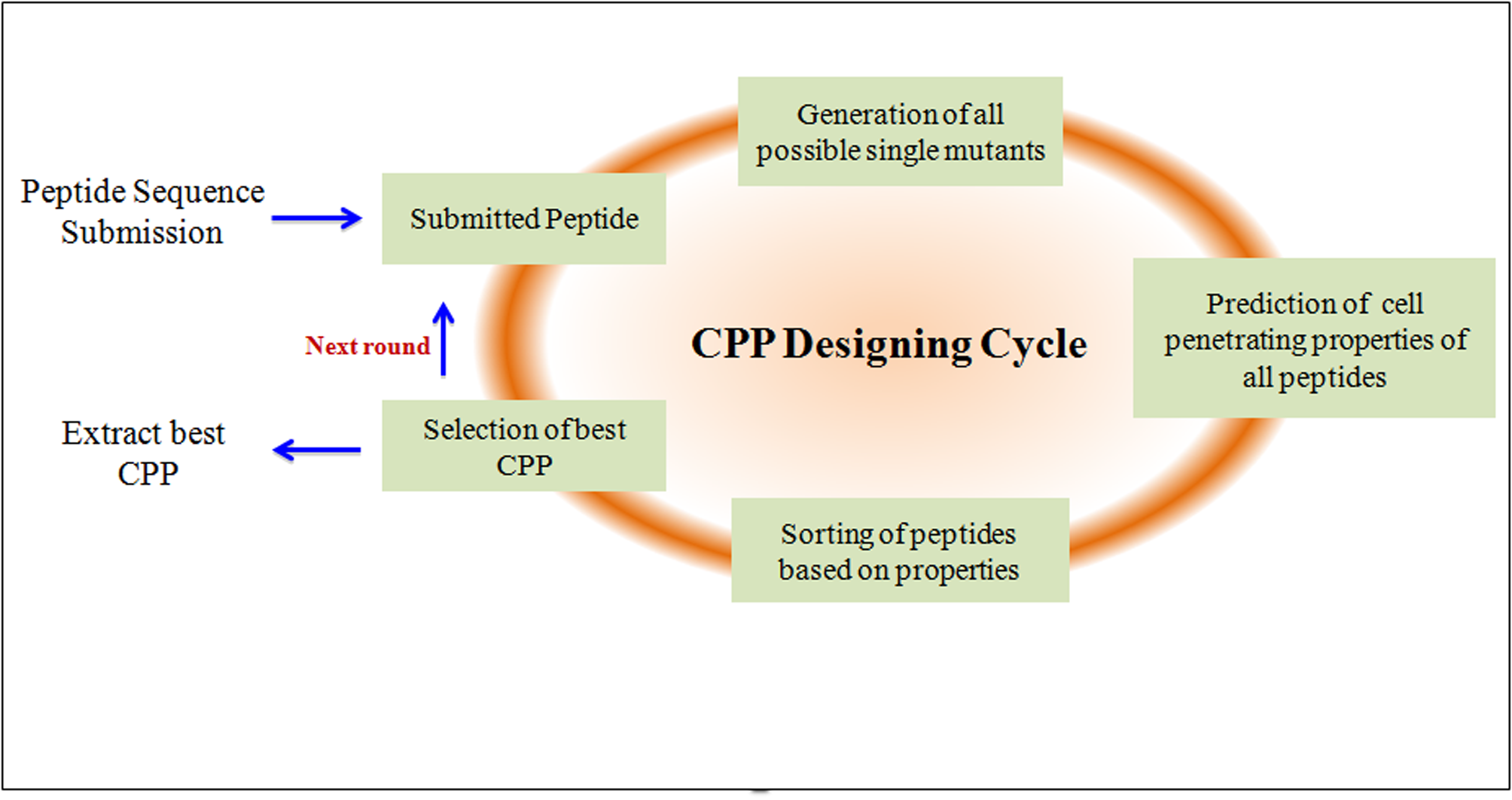


**Figure 1.** CPP designing cycle showing various steps for designing CPP analouges using CellPPD.

**Example 1: Designing of best cell penetrating analogues based on a random sequence RRGIRLWSHLPRK**

User can follow the below steps in order to design better cell penetrating analogues.

***Step 1. Submission of RRGIRLWSHLPRK***

Go to **“Design peptide”** tool and type the peptide sequence in single letter code as described in following Figure 2.

There are two options for prediction, one is SVM based and other is SVM + motif based.

User can select both options one by one as per the convenience. For the prediction, user has to select SVM threshold and E-value cut-off for SVM based and motif based method, respectively. As this server allows users to select a threshold, we suggest the users to select higher value, if they are interested in high specificity (high confidence). In addition, several physicochemical properties like hydrophobicity, amphipathicity, pI, charge, *etc*. can be selected to be displayed along with prediction status. For example, we choose SVM method for prediction with threshold 0.3.


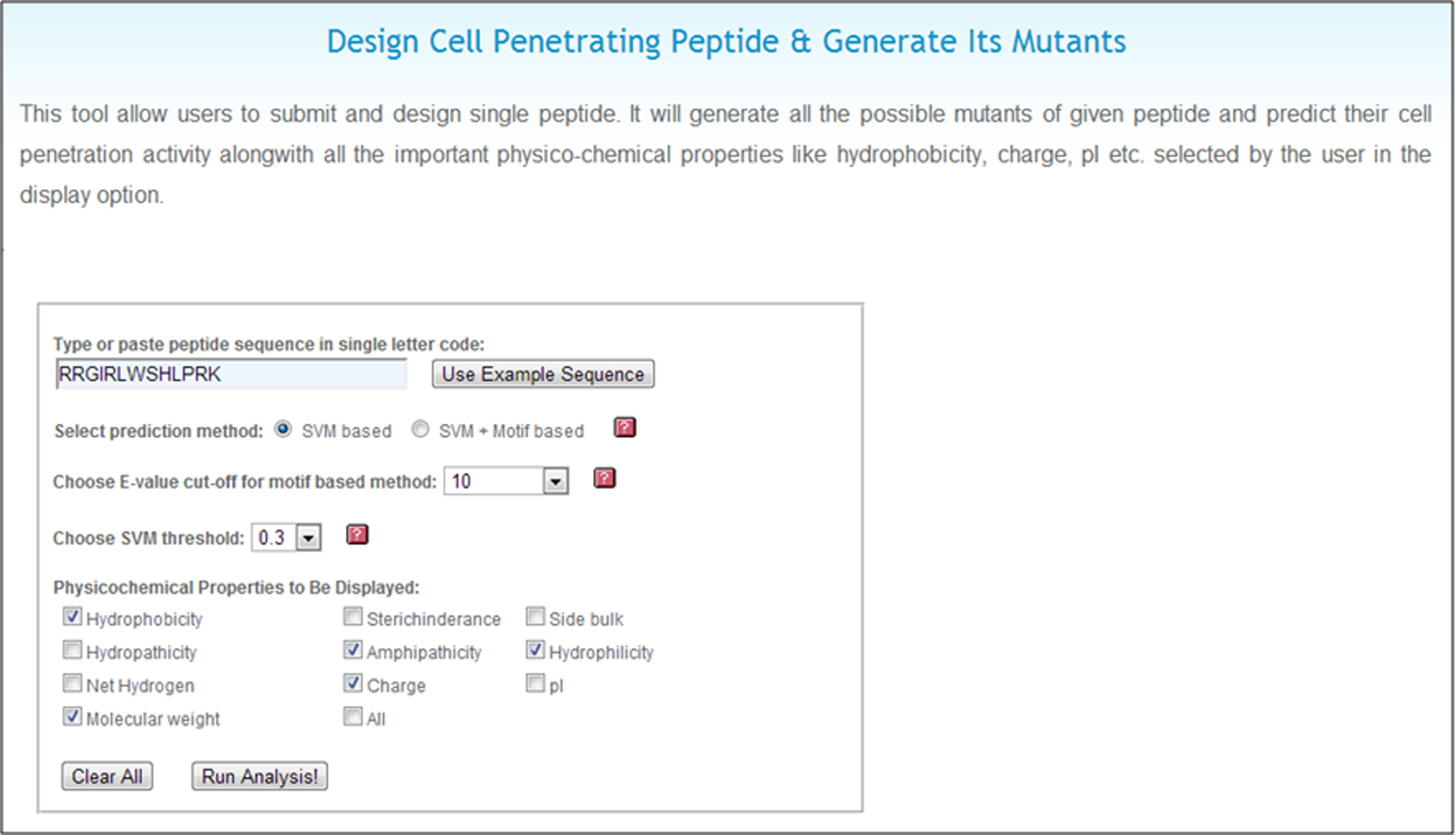


**Figure 2.** Submission page for design peptide tool.

***Step 2. Prediction of submitted peptide and it’s all possible mutants.***

Server predicted the submitted peptide as CPP with SVM score 0.4 (Figure 3).


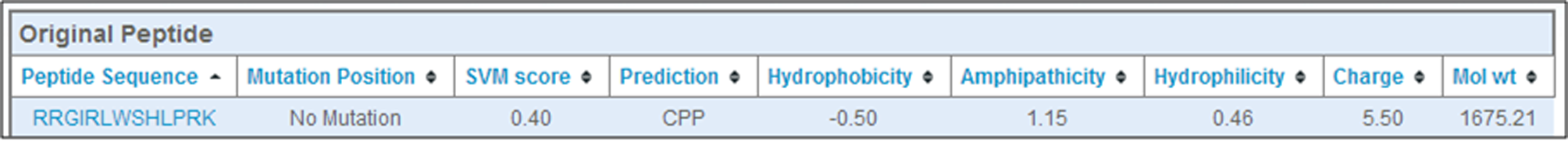


**Figure 3.** Result of SVM-based prediction.

In addition to prediction of original peptide, server also generates all possible single substitution mutants (depicted in red color) of the original peptide with their SVM score and prediction status (Figure 4). Various physicochemical properties have also displayed.


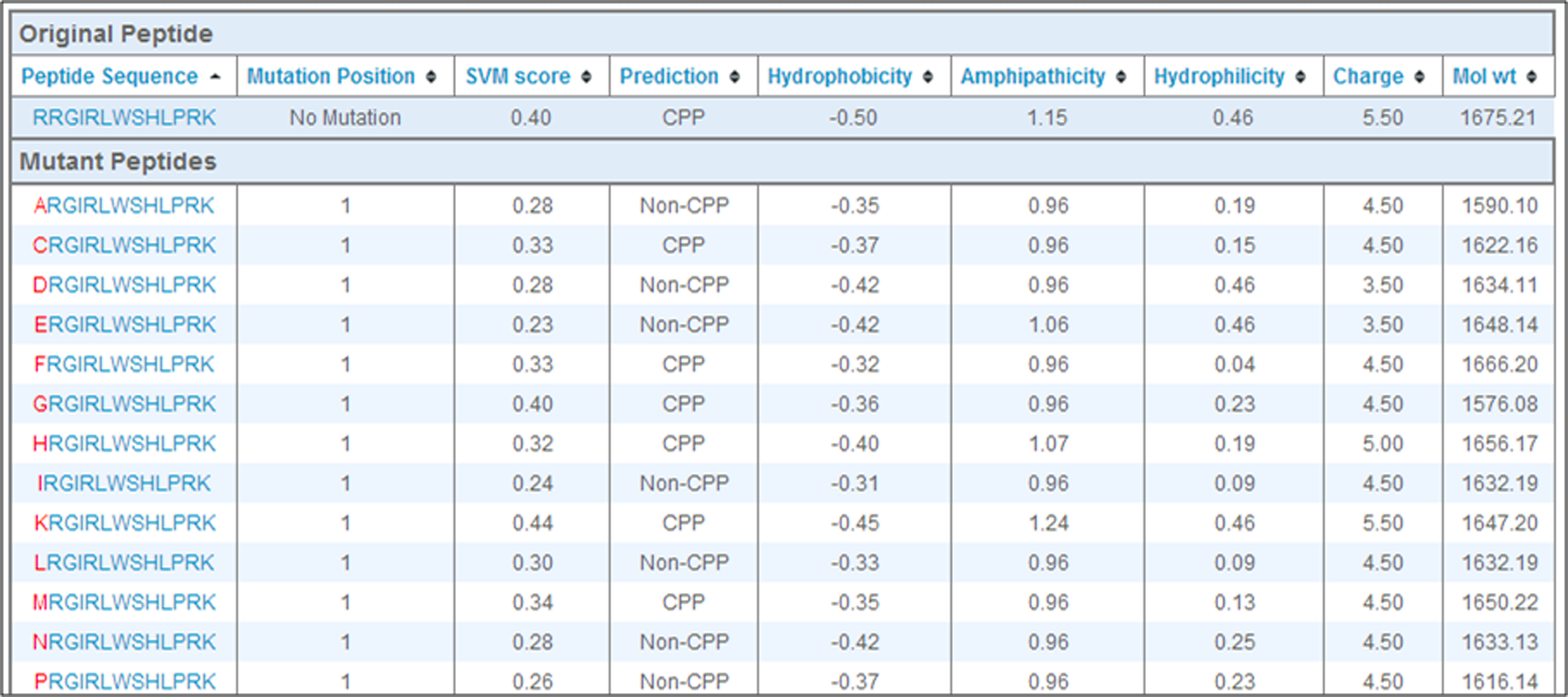


**Figure 4.** A screenshot of complete result of SVM-based prediction showing all possible mutants with their physicochemical properties, SVM score and prediction status.

***Step 3. Selection of best cell penetrating analogue with desired properties.***

In CellPPD, sorting options for all the properties have been provided. User can sort peptide analogues having desired SVM score and physicochemical properties. For example, we have sorted analogues according to their SVM scores to select analouges having higher SVM score (Figure 5) than the original peptide. After sorting, analogue RRGRRLWSHLPRK displayed the highest SVM score (0.63) amongst all analogues and original peptide. Similarly, user can sort other properties as well.

**
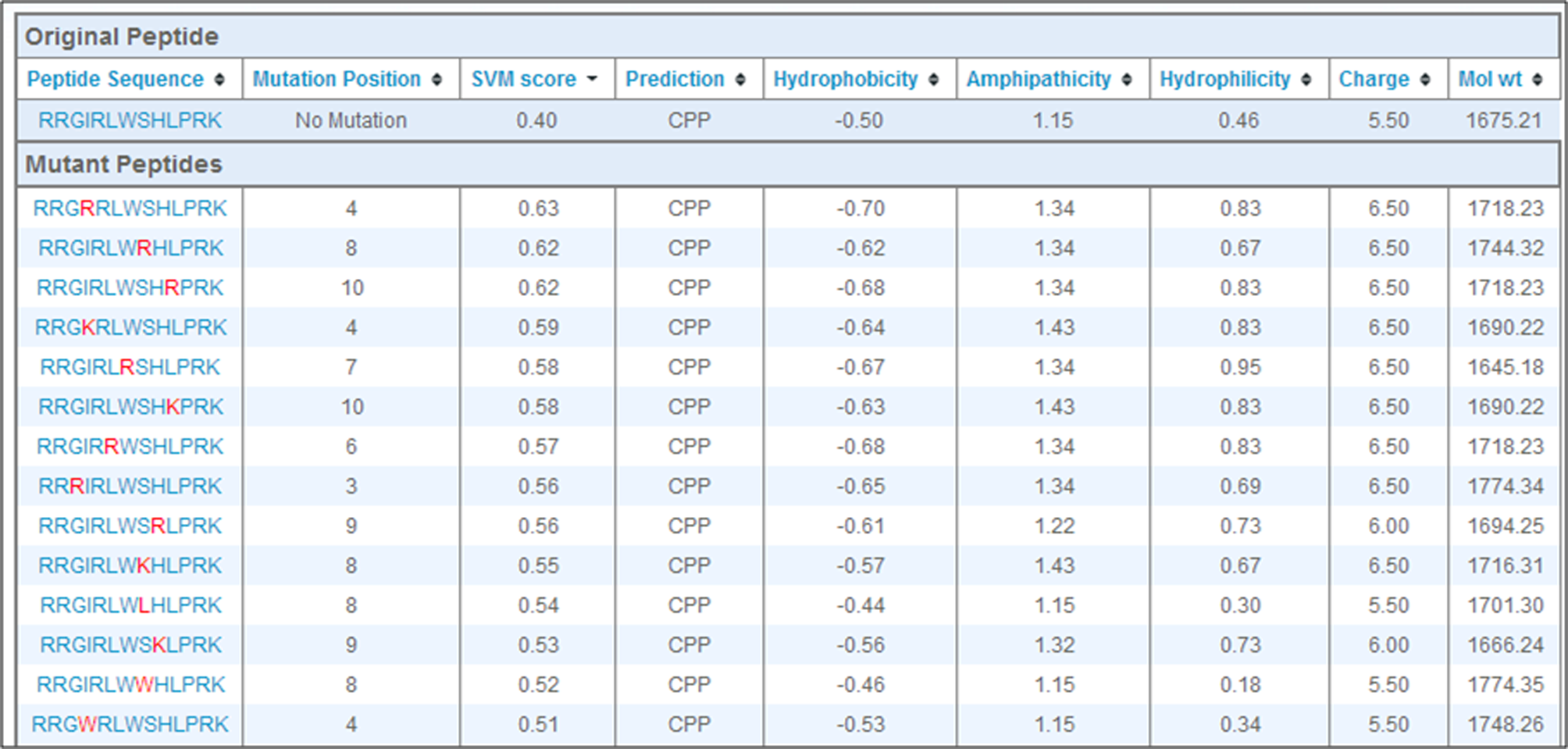
**

**Figure 5.** Sorting of results obtained in step 2.

***Step 4. Generation of further all possible mutants of selected analogue (RRGRRLWSH LPRK) with prediction status.***

User can further generate all possible mutants of their desired analogues obtained in step 3 by clicking on the peptide. For example, we have selected and clicked on RRGRRLWSHLPRK analogue, and server generated further all possible mutants of the RRGRRLWSHLPRK with SVM score, prediction status and all physicochemical properties (Figure 6). User can again sort the obtained results and select best cell penetrating analogue (based on SVM score and physicochemical properties). This cycle can be run until best cell penetrating analogue is obtained.


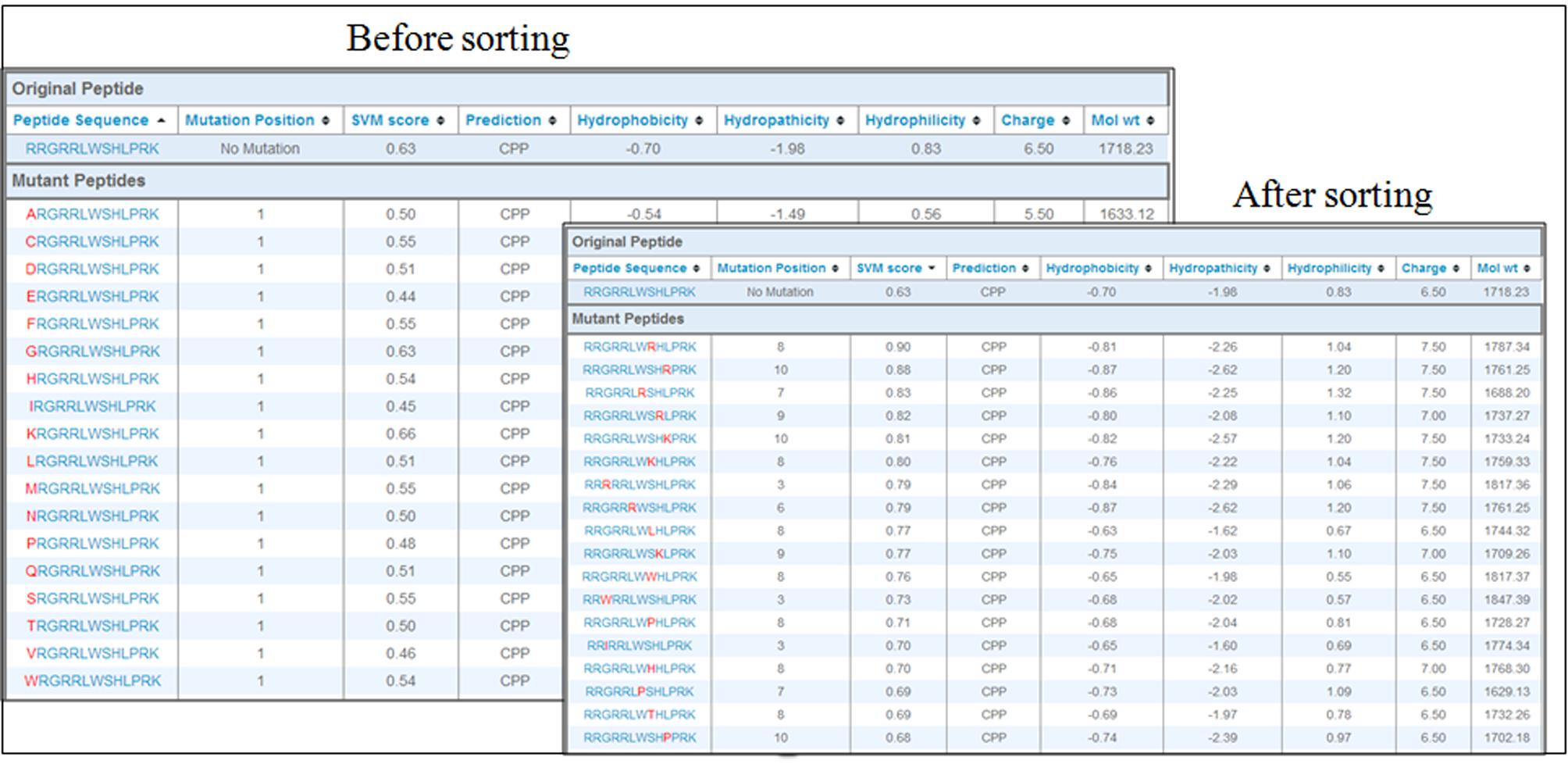


**Figure 6.** All possible mutants with their SVM score and prediction status of RRGRRLWSHLPRK peptide.

**Example 2. Designing of CPPs from a protein sequence.**

Since most of the existing CPPs are derived from natural proteins. CellPPD provides facility to identify potential CPPs from a protein sequence. A tool **protein scanning** has been implemented to web server for the detection of putative CPPs in a protein sequence. In this tool, after submission of a query protein sequence, server first generates overlapping peptides of window length selected by the user, where all the peptides will be clickable. All peptides are then predicted by the server and presented in tabular format with their SVM score and prediction status. User can select the best CPPs based on SVM score. In addition, CellPPD also generates all possible single substitution mutants of the selected peptide with their SVM score and prediction status in a similar manner described above. The overall approach is demonstrated in Figure 7.


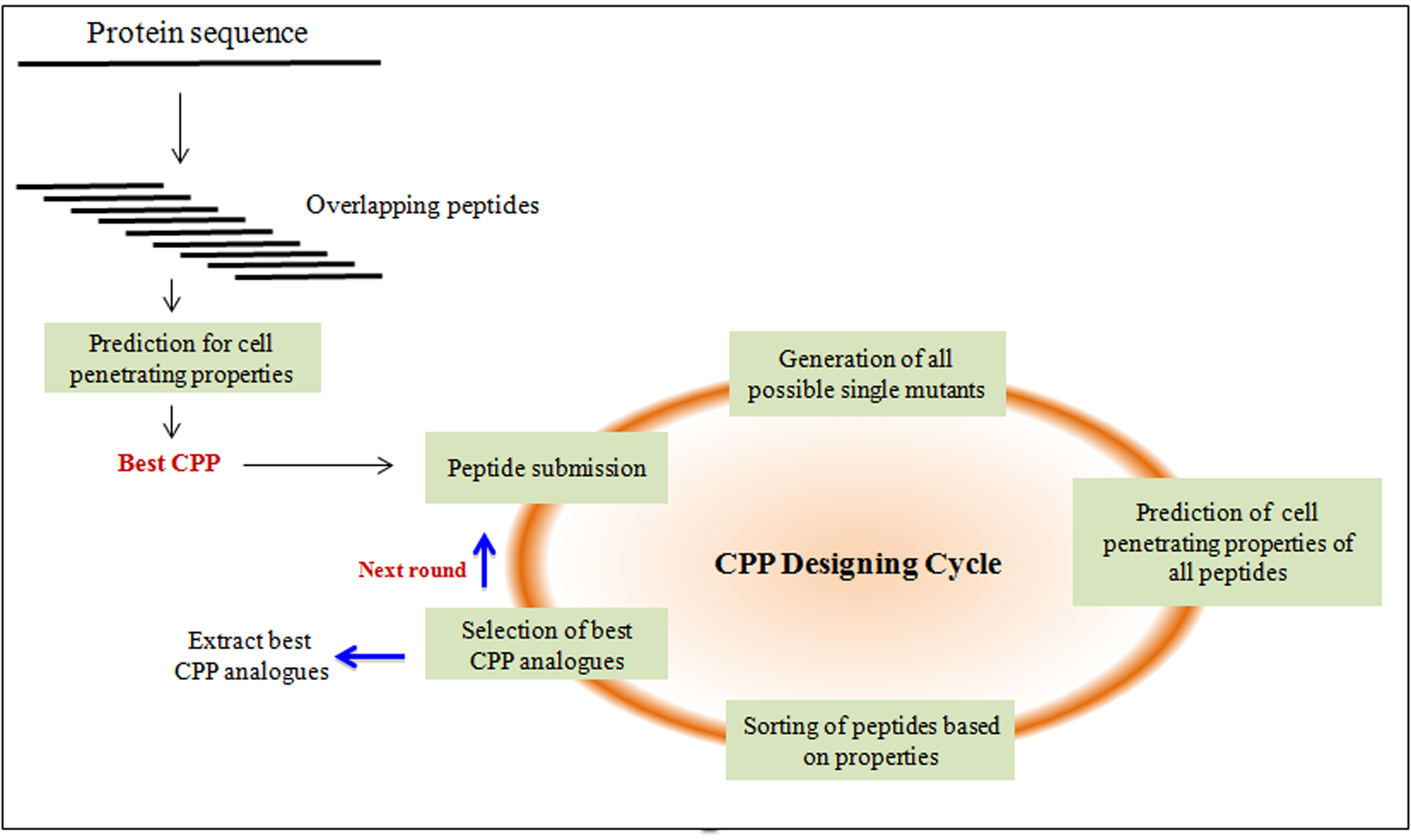


**Figure 7.** Designing of CPP by protein scanning tool.

For example, we wish to identify putative CPP sequences in the following protein sequence

AGLQFPVGRVHRLLRDSDCPGACICFACRICMRNFSTRQARRNHRRRHGVCPKILKKCRCSIRICMRRDSDCPGACICRGNGYCGSGWTLNSAGYLLGKINLKALAALAKKRQIKIWFQNRRMKWKK

An option for selection of peptide length has provided. We have chosen the peptide length 10 and SVM based method with threshold 0.7 for the prediction of CPPs (Figure 8). User can select either options (SVM based or Motif based) for the prediction.


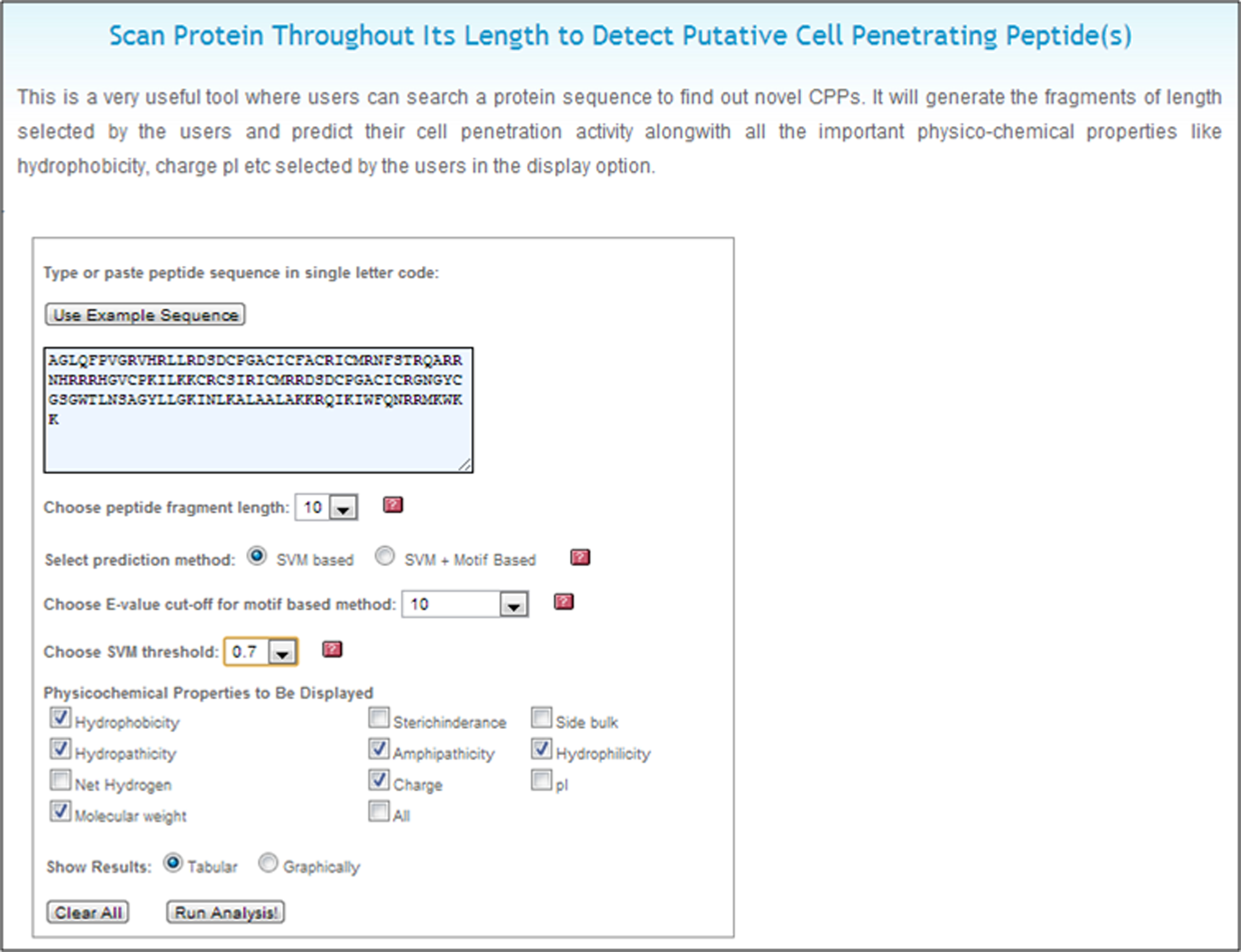


**Figure 8.** Submission page of protein scanning page.

Server generated overlapping peptides of window length 10 with their SVM score and prediction status, where all the peptides are clickable (Figure 9).


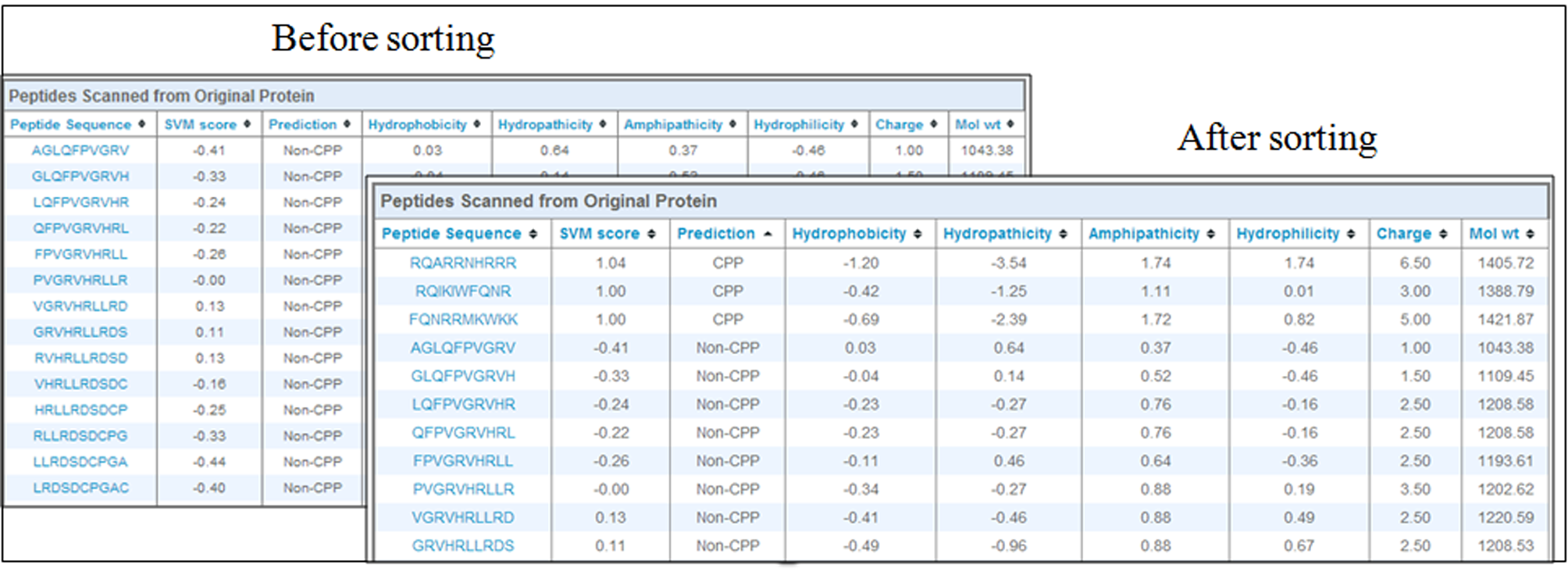


**Figure 9.** Screenshot of SVM based result of submitted protein sequence showing all possible peptides (length10) with their physicochemical properties, SVM scores and prediction status.

In protein scanning tool, user can also obtain their results in graphical format (Figure 10), where all the values *e.g.* SVM and other physicochemical properties can be plotted.


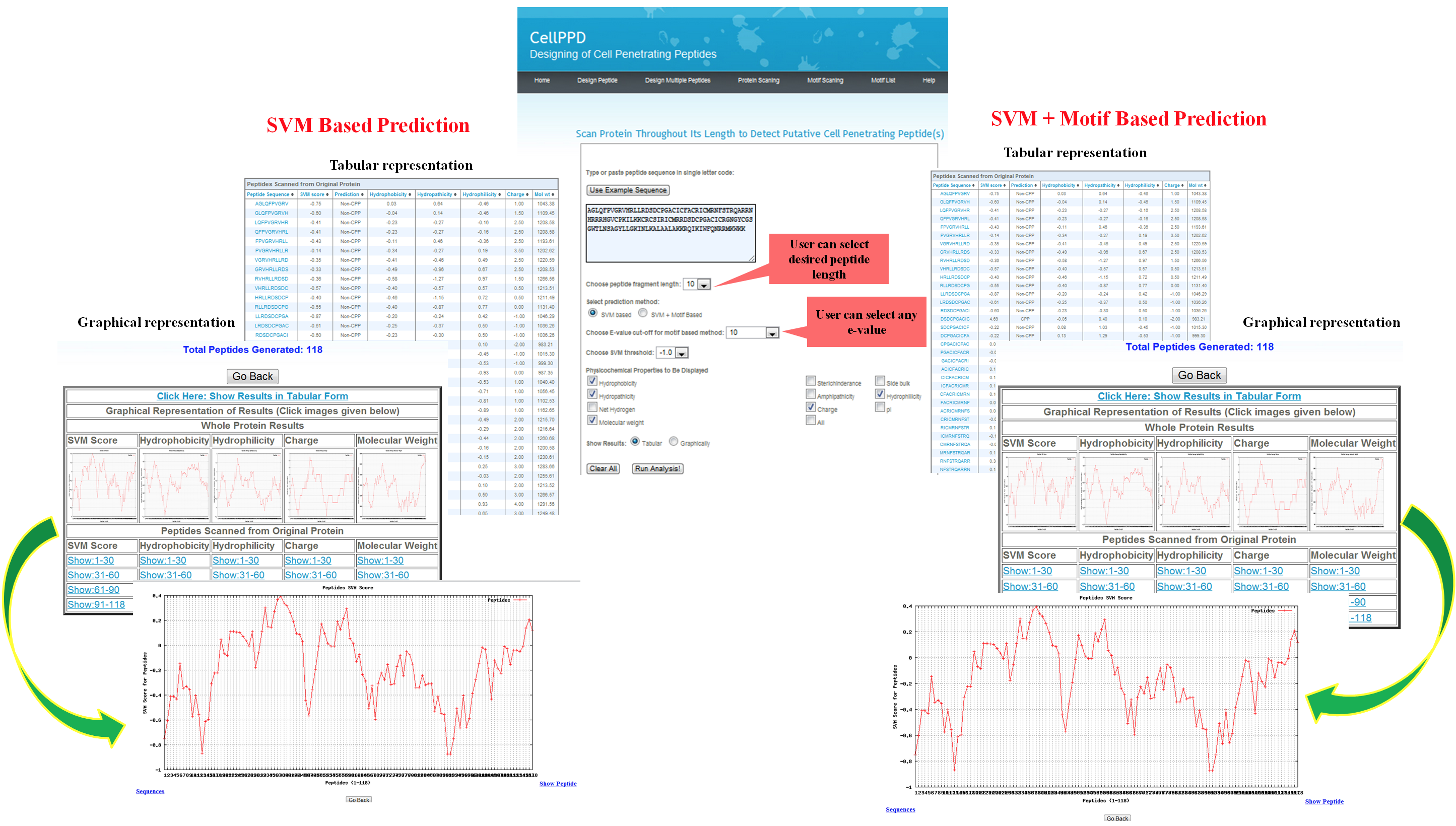


**Figure 10**. Schematic representation of SVM results in graphical format.

**Case studies for accuracy comparison of CellPPD**

In order to help the user and accuracy comparison, we have carried out some tests with well-known CPPs and their non-CPP analogues. We have checked the prediction of these peptides with CellPPD web server.

**Example 1.** *p*VEC and scrambled *p*VEC

*p*VEC is a well-known CPP derived from the murine vascular endothelial-cadherin protein, while its analogue scrambled *p*VEC, is not a CPP (Elmquist *et al*. 2006). We have performed the prediction of these peptides using CellPPD at threshold 0.5. The results are as follows:

| **Peptide name** | **Sequence** | **SVM Score** | **Prediction** |
| --- | --- | --- | --- |
| *p*VEC | LLIILRRRIRKQAHAHSK | 1.27 | CPP |
| Scramble *p*VEC | IAARIKLRSRQHIKLRHL | 0.12 | Non-CPP |

CellPPD successfully discriminates both peptides in two classes.

**Example 2. Penetratin and non- penetrating Penetratin analogues.**

Penetratin is a well-known CPP derived from the third α-helix of the homeodomain (residues 43-58). Various analogues of Penetratin have been found to be non-CPP (Fischer *et al*. 2000). We have performed the prediction of these peptides using CellPPD. The results are as follows:

| **Peptide name** | **Sequence** | **SVM Score** | **Prediction** |
| --- | --- | --- | --- |
| Penetratin (43-58) | RQIKIWFQNRRMKWKK | 1.32 | CPP |
| Non-CPP analog 1  (2Phe) | RQIKIFFQNRRMKFKK | 1.03 | CPP |
| Non-CPP analog 2  (46-60) | KIWFQNRRMK | 0.47 | Non-CPP |
| Non-CPP analog 3  (41-55) | TERQIKIWFQNRRMK | 0.07 | Non-CPP |

CellPPD successfully predicted Penetratin peptide as CPP and two non-penetrating analogues of Penetratin as non-CPPs. Only analogue 1 (2Phe) is predicted as CPP. Analog 1 is a double substitution mutant (Trp/Phe) of Penetratin, and highly similar to Penetratin (different by only two residues from Penetratin). However, there is a significant difference between the SVM score (from 1.32 to 1.03), which suggests that besides significant similarity, CellPPD can differentiate this analogue from Penetratin.
